# Effects of aerobic exercise on hippocampal formation volume in people with schizophrenia – a systematic review and meta-analysis with original data from a randomized-controlled trial

**DOI:** 10.1017/S0033291724001867

**Published:** 2024-11

**Authors:** Lukas Roell, Tim Fischer, Daniel Keeser, Boris Papazov, Moritz Lembeck, Irina Papazova, David Greska, Susanne Muenz, Thomas Schneider-Axmann, Eliska Sykorova, Cristina E. Thieme, Bob O. Vogel, Sebastian Mohnke, Charlotte Huppertz, Astrid Roeh, Katriona Keller-Varady, Berend Malchow, Sophia Stoecklein, Birgit Ertl-Wagner, Karsten Henkel, Bernd Wolfarth, Wladimir Tantchik, Henrik Walter, Dusan Hirjak, Andrea Schmitt, Alkomiet Hasan, Andreas Meyer-Lindenberg, Peter Falkai, Isabel Maurus

**Affiliations:** 1 Department of Psychiatry and Psychotherapy, LMU University Hospital, LMU Munich, Munich, Germany; 2 Neuroimaging Core Unit Munich (NICUM), LMU University Hospital, LMU Munich, Munich, Germany; 3 Munich Center for Neurosciences (MCN), LMU Munich, Munich, Germany; 4 Department of Radiology, LMU University Hospital, LMU Munich, Munich, Germany; 5 Department of Psychiatry, Psychotherapy and Psychosomatics of the University Augsburg, Medical Faculty, University of Augsburg, Bezirkskrankenhaus Augsburg, Augsburg, Germany; 6 Medical Faculty Mannheim, Central Institute of Mental Health, Heidelberg University, Heidelberg, Germany; 7 Department of Psychiatry and Psychotherapy, University Hospital Charité Berlin, Berlin, Germany; 8 Department of Psychiatry, Psychotherapy and Psychosomatics, RWTH Aachen University, Aachen, Germany; 9 Department of Rehabilitation and Sports Medicine, Hannover Medical School, Hannover, Germany; 10 Department of Psychiatry and Psychotherapy, University Hospital Göttingen, Göttingen, Germany; 11 Division of Neuroradiology, Department of Diagnostic Imaging, The Hospital for Sick Children, Toronto, Canada; 12 Department of Medical Imaging, University of Toronto, Toronto, Canada; 13 Department of Sports Medicine, University Hospital Charité Berlin, Berlin, Germany; 14 German Center for Mental Health (DZPG), partner site Mannheim/Heidelberg/Ulm, Germany; 15 Laboratory of Neuroscience (LIM27), Institute of Psychiatry, University of Sao Paulo, São Paulo, Brazil; 16 Max Planck Institute of Psychiatry, Munich, Germany; 17 German Center for Mental Health (DZPG), partner site Munich/Augsburg, Germany

**Keywords:** exercise, hippocampus, meta-analysis, randomized-controlled trial, schizophrenia, volume

## Abstract

**Background:**

The hippocampal formation represents a key region in the pathophysiology of schizophrenia. Aerobic exercise poses a promising add-on treatment to potentially counteract structural impairments of the hippocampal formation and associated symptomatic burden. However, current evidence regarding exercise effects on the hippocampal formation in schizophrenia is largely heterogeneous. Therefore, we conducted a systematic review and meta-analysis to assess the impact of aerobic exercise on total hippocampal formation volume. Additionally, we used data from a recent multicenter randomized-controlled trial to examine the effects of aerobic exercise on hippocampal formation subfield volumes and their respective clinical implications.

**Methods:**

The meta-analysis comprised six studies that investigated the influence of aerobic exercise on total hippocampal formation volume compared to a control condition with a total of 186 people with schizophrenia (100 male, 86 female), while original data from 29 patients (20 male, 9 female) was considered to explore effects of six months of aerobic exercise on hippocampal formation subfield volumes.

**Results:**

Our meta-analysis did not demonstrate a significant effect of aerobic exercise on total hippocampal formation volume in people with schizophrenia (*g* = 0.33 [−0.12 to 0.77]), *p* = 0.15), but our original data suggested significant volume increases in certain hippocampal subfields, namely the cornu ammonis and dentate gyrus.

**Conclusions:**

Driven by the necessity of better understanding the pathophysiology of schizophrenia, the present work underlines the importance to focus on hippocampal formation subfields and to characterize subgroups of patients that show neuroplastic responses to aerobic exercise accompanied by corresponding clinical improvements.

**Trial Registration:**

The meta-analysis was pre-registered on the international Prospective Register of Systematic Reviews (PROSPERO) (2023 CRD42023426953).

The randomized-controlled trial on which the current analysis is based on was registered in the International Clinical Trials Database, ClinicalTrials.gov (NCT number: NCT03466112, https://clinicaltrials.gov/ct2/show/NCT03466112?term=NCT03466112&draw=2&rank=1) and in the German Clinical Trials Register (DRKS-ID: DRKS00009804).

**Data Set Information:**

Details on the study design and data set are available under the following link: 10.1007/s00406-020-01175-2.

## Introduction

The hippocampal formation (HF) is a key brain region that consists of several subfields such as the cornu ammonis (CA1–4), the dentate gyrus (DG) or the subiculum complex (Schultz & Engelhardt, 2014). The HF plays a fundamental role in long-term episodic, semantic and short-term memory as well as spatial processing (Bird & Burgess, 2008). In this context, the HF subregions contribute differently to particular domains of memory functioning (Duncan, Ketz, Inati, & Davachi, 2012; Eldridge, Engel, Zeineh, Bookheimer, & Knowlton, 2005; Hainmueller & Bartos, 2020; Zeineh, Engel, Thompson, & Bookheimer, 2003).

In schizophrenia, volume reductions in the HF represent a consistent endophenotype (Adriano, Caltagirone, & Spalletta, 2012; Brugger & Howes, 2017; Chopra et al., 2023; Honea, Crow, Passingham, & Mackay, 2005; van Erp et al., 2016), with a particularly pronounced decline in the CA1–4, DG, and subiculum (Falkai et al., 2016; Haukvik, Tamnes, Söderman, & Agartz, 2018; Ho et al., 2017; Mathew et al., 2014; Nakahara et al., 2020; Nakahara, Matsumoto, & van Erp, 2018; Roeske, Konradi, Heckers, & Lewis, 2021; Schmitt et al., 2022). Decreases of both global and subfield volumes have been associated with impairments in numerous cognitive domains such as verbal short- and long-term memory, and processing speed (Antoniades et al., 2018; Haukvik et al., 2018; Khalil, Hollander, Raucher-Chéné, Lepage, & Lavigne, 2022; Nakahara et al., 2018, 2020; Pijnenborg et al., 2020) as well as with increased severity of positive and negative symptoms (Haukvik et al., 2018; Ho et al., 2017; Nakahara et al., 2018).

In the last two decades, aerobic exercise interventions have been increasingly proposed as a promising add-on treatment to improve psychopathological symptoms and cognitive functioning in psychiatric disorders (Schmitt, Reich-Erkelenz, Hasan, & Falkai, 2019). Relevant large-scale evidence indicates that different types of exercise can improve psychiatric symptoms (Dauwan, Begemann, Heringa, & Sommer, 2016; Fernández-Abascal, Suárez-Pinilla, Cobo-Corrales, Crespo-Facorro, & Suárez-Pinilla, 2021; Firth, Cotter, Elliott, French, & Yung, 2015; Gallardo-Gómez et al., 2023; Guo, Liu, Liao, Qin, & Yue, 2024; Kim, Lee, & Kang, 2023; Maurus et al., 2023; Sabe, Kaiser, & Sentissi, 2020; Vogel et al., 2019; Wei et al., 2020; Ziebart et al., 2022), cognition (Fernández-Abascal et al., 2021; Firth et al., 2015; Shimada et al., 2022; Xu et al., 2022) and overall functioning (Dauwan et al., 2016; Fernández-Abascal et al., 2021; Korman et al., 2023) in people with schizophrenia, as is particularly the case for aerobic exercise interventions (Firth et al., 2017b; Sabe et al., 2020; Shimada et al., 2022; Vogel et al., 2019; Xu et al., 2022).

Beneficial effects of aerobic exercise on clinical health outcomes related to schizophrenia may be driven by improvements in physical health (Vancampfort, Rosenbaum, Ward, & Stubbs, 2015) and by multiple neural adaptations that occur in response to aerobic exercise treatments (Firth, Cotter, Carney, & Yung, 2017a; Maurus et al., 2019). Specifically, aerobic exercise interventions in people with schizophrenia have been found to increase the volume in the HF and its subfields, which is accompanied by improvements in short-term memory, working memory and overall functioning (Falkai et al., 2021; Khonsari et al., 2022; Lin et al., 2015; Pajonk et al., 2010; Woodward et al., 2018). However, the body of literature as a whole remains heterogeneous, as several studies also report null-results in both patients with manifested schizophrenia (Malchow et al., 2016; Rosenbaum et al., 2015; Scheewe et al., 2013) and people at clinical high-risk for psychosis (Damme et al., 2022; Dean et al., 2017). Moreover, only a few studies included the HF subfields in their respective analyses despite their potential clinical relevance in schizophrenia (Damme et al., 2022; Malchow et al., 2016; Woodward et al., 2018).

In order to summarize the current heterogeneous state of research, we conducted a systematic review and meta-analysis and analyzed data from our recent multicenter randomized-controlled exercise study in people with schizophrenia. First, we examined if aerobic exercise increases global hippocampal formation volume in schizophrenia. We addressed said question by means of synthesizing existing literature and our present findings in a meta-analysis. Second, based on our current study, we investigated if certain HF subfield volumes are particularly responsive to aerobic exercise. Third, we explored if increases in individual HF subfield volumes after six months of aerobic exercise are related to changes in clinical symptom severity, cognitive performance, and levels of general functioning.

## Methods and materials

### Meta-analysis

#### Search strategy

Our meta-analysis was pre-registered on the international Prospective Register of Systematic Reviews (PROSPERO) (2023 CRD42023426953). Preferred Reporting Items for Systemic Reviews and Meta-Analyses (PRISMA) standards were adhered to throughout the review process. Using the following keywords: ‘Schizophrenia’ OR ‘Psychosis’ AND ‘Aerobic exercise’ OR ‘Physical activity’ OR ‘Fitness’ AND ‘Hippocampus’ OR ‘Hippocampal volume’, TF and LR searched the ensuing databases: Cochrane, PubMed, Web of Science, ISRCTN, and Embase from inception until 25th April 2023 for published and unpublished literature. After removal of duplicates, titles and abstracts were reviewed independently by TF and LR using Rayyan software (Ouzzani, Hammady, Fedorowicz, & Elmagarmid, 2016). Full-text papers were identified according to pre-specified eligibility criteria and primary outcome. In case of disagreement, a third reviewer (IM) was available to mediate. Data extraction began on 26th May 2023.

#### Eligibility criteria

Inclusion criteria comprised studies that: (1) focused on schizophrenia spectrum disorder, (2) included an aerobic exercise intervention at moderate intensity, e.g. bicycle ergometer, treadmill or similar, (3) included a control condition that does not amount to aerobic exercise, e.g. waitlists, table soccer, (4) reported total hippocampal formation volume assessed via structural magnetic resonance imaging and analyzed by means of manual or automated segmentation. Moreover, it was required that studies were published in English language and included an adult participant sample 18+ years of age.

#### Risk of bias and quality assessment

The most recent edition of the Cochrane risk-of-bias tool for randomized trials (RoB 2) was used to assess risk of bias (Sterne et al., 2019). The tool encompasses five domains: randomization process, deviations from intended interventions, missing outcome data, measurement of the outcome, and selection of the reported result. Based on answers to signaling questions, an algorithm determined the extent of bias relevant to a particular domain as well as an overall bias score ranging from ‘low risk of bias’ to ‘some concerns’ and ‘high risk of bias’ (online Supplementary Figs S1 and S2). Both TF and LR completed the risk of bias assessment using the RoB 2.0 excel template. Inconsistencies were resolved by means of discussion.

#### Data extraction

Our primary outcome was the change in total hippocampal formation volume from pre- to post-intervention. The following data were extracted independently from included reports by TF and LR: sample sizes, means and standard deviations of primary outcome data prior to and post aerobic exercise interventions for the experimental and control groups. If said data were not made available in the initial report, authors were contacted by either TF or LR.

### Statistical analysis

Comprehensive meta-analysis software (V4) was used to conduct random-effects meta-analyses (Borenstein, 2022) based on all trials with control condition comparing the effects on hippocampal formation volume in an aerobic exercise intervention to a study-specific control group. Hedges' *g* statistic was computed using intervention effect sizes (differences between intervention and control groups) for the primary outcome measurement, as were 95% confidence intervals (CIs). Effect sizes expressed as Hedges' *g* were classified as small (0.2 ⩽ *g* < 0.5), medium (0.5 ⩽ *g* < 0.8), or large (*g* > 0.8). A sensitivity analysis was conducted by means of removing one study at a time in order to assess robustness of observed effects. Heterogeneity was assessed using Cochran's *Q* and Higgins' *I^2^*, which can be divided into considerable (>75%), substantial (50–75%), moderate (30–50%), and low (< 30%). Publication bias was addressed via producing a funnel plot and assessing asymmetry using Egger's regression coefficient as well as determining Begg and Mazumdar's rank correlation value with *p* < 0.05 indicative of bias (Begg & Mazumdar, 1994; Egger, Davey Smith, Schneider, & Minder, 1997). In case of significant publication bias, a Duval and Tweedie trim-and-fill analysis was utilized (Duval & Tweedie, 2000). Moreover, in exploratory intention, we conducted a second meta-analysis which included studies without control conditions (Rosenbaum et al., 2015; Woodward et al., 2018) and underaged participants (Dean et al., 2017). This analysis was based on all available studies comparing the HF volume prior to the aerobic exercise intervention with the volume after the aerobic exercise intervention. The rationale behind the exploratory analysis was the limited number of studies surrounding the effect of aerobic exercise compared to a control condition on HF volume in schizophrenia and the low risk of placebo effects when investigating HF volume as a standalone outcome.

### Randomized-controlled trial

#### Study design and sample

The data presented here originate from the Enhancing Schizophrenia Prevention and Recovery through Innovative Treatments (ESPRIT) C3 study (NCT03466112, https://clinicaltrials.gov/ct2/show/NCT03466112?term=NCT03466112&draw=2&rank=1). The ESPRIT C3 study is a multicenter randomized-controlled trial that investigates the effects of aerobic exercise on multiple health outcomes in people with schizophrenia. The authors assert that all procedures contributing to this work comply with the ethical standards of the relevant national and institutional committees on human experimentation and with the Helsinki Declaration of 1975, as revised in 2008. All procedures involving human patients were approved by the ethics committee of the medical faculty of the Ludwig-Maximilians-University Munich (approval number: 706-15).

In total 180 participants gave written informed consent and were randomly assigned to either an aerobic endurance training (AET) or to a flexibility, strengthening and balance training (FSBT). Supervised by a sport scientist, both groups exercised up to three times per week, for approximately 40–50 min per session, for a total duration of six months. Patients in the AET group cycled on a stationary bicycle ergometer at a moderate exercise intensity which was determined by a lactate threshold test prior to the onset of the intervention. In the FSBT group, patients performed exercises covering stretching, mobility, stability, balance, and relaxation (Liu-Ambrose et al., 2010). Ninety nine subjects initially agreed to undergo MRI sessions, but evaluable structural T1-weighted MR images from which HF subfield volumes could be computed at baseline and post-intervention were available for only 29 participants (9 in the AET group, 20 in the FSBT group). Table 1 depicts the sample characteristics. Details of the ESPRIT C3 study are described in the supplemental information and in the corresponding study design publication (Maurus et al., 2020).

**Table 1.** Sample characteristics

	AET *n* = 9 (31.0%) (*n*/mean ± s.d.)	FSBT *n* = 20 (51.6%) (*n*/mean ± s.d.)	
Study site			*p*_fisher_ = 0.161
Munich	6 (66.7%)	17 (85.0%)	
Mannheim	3 (33.3%)	1 (5.0%)	
Berlin	0 (0.0%)	2 (10.0%)	
Sex			*p*_fisher_ = 1.000
Female	3 (33.3%)	6 (30.0%)	
Male	6 (66.7%)	14 (70.0%)	
Age (years**)**	34.67 ± 12.29	39.40 ± 11.25	*p*_wilcox_ = 0.299
PANSS at baseline (t0)			
Positive	10.22 ± 3.03	10.65 ± 3.70	*p*_wilcox_ = 1.000
Negative	11.56 ± 4.95	12.10 ± 3.60	*p*_wilcox_ = 0.477
Total	45.44 ± 12.93	46.45 ± 9.42	*p*_wilcox_ = 0.539
Chlorpromazine equivalents	291.96 ± 151.00	438.23 ± 246.76	*p*_wilcox_ = 0.150
Education (years)	12.33 ± 3.35	16.25 ± 4.84	*p*_wilcox_ = 0.064
Total number of trainings	41.11 ± 16.34	35.70 ± 18.25	*p*_wilcox_ = 0.396

AET, aerobic endurance training; FSBT, flexibility, strengthening and balance training; PANSS, Positive and Negative Syndrome Scale; p_fisher_, *p* value of Fisher's exact test for categorical data; *p*_wilcox_, *p* value of Wilcoxon signed-rank test for numeric data; s.d., standard deviation. The sample sizes per group refer to the number of participants that were considered for the statistical data analysis.

#### Structural MRI data and clinical variables

The hippocampal module of FreeSurfer v7.2 (Iglesias et al., 2015) was used to compute the volumes of HF subfields at a spatial isotropic resolution of 0.8 mm^3^ at study site Munich and 1 mm^3^ at study sites Mannheim and Berlin. Based on the proportion method (O'Brien et al., 2011), HF volumes were corrected via intracranial volume across all subjects and sessions and volumes of bodies and heads were summed up for each subfield. Because the FreeSurfer segmentation of all HF subfields based on MRI scans at a spatial isotropic resolution of 1 mm^3^ has been criticized due to issues regarding the detection of the boundaries between subfields (Wisse et al., 2021), we conducted an additional analysis segmenting the HF only into head, body, and tail.

The Positive and Negative Syndrome Scale (PANSS) was administered to assess positive and negative symptoms, and total symptom severity (Kay, Fiszbein, & Opler, 1987). Verbal semantic long-term memory comprised a composite score of the sixth and seventh run of the Verbal Learning and Memory Test (VLMT), whereas the average of the first and interference run was utilized to cover verbal semantic short-term memory (Helmstaedter & Durwen, 1990). The backward version of the Digit Span Test (DST) was employed to target verbal working memory (Tewes, 1994). The Global Assessment of Functioning scale (GAF) and the Functional Remission of General Schizophrenia scale (FROGS) were applied to assess general functioning (Endicott, Spitzer, Fleiss, & Cohen, 1976; Llorca et al., 2009). Further details on MRI data acquisition (online Supplementary Table S2), processing, and quality control procedures, as well as on the cognitive test batteries are provided in the supplemental information.

### Statistical analysis

Statistical analysis was performed with Rstudio v1.4.1717 based on R v4.2.2 (R Core Team, 2021; RStudio Team, 2020) and visualizations were created with *ggplot2* (Wickham, 2016).

To investigate the effect of AET on HF subfield volumes in comparison to FSBT (2nd research question), linear mixed effect models for repeated measures were calculated. Group (AET, FSBT), time (t0: baseline, t6: six months), the group × time interaction, age, sex, chlorpromazine equivalents, number of trainings, volume at baseline, study site (Munich, Mannheim, Berlin), and hemisphere (left, right) were included as predictors, while the corresponding five subfield volumes served as dependent variables. *p* values of the factor time and the group × time interaction were adjusted separately across the five linear mixed models using the false discovery rate (FDR) method (Benjamini & Hochberg, 1995). In case of a significant group × time interaction (*p*_fdr_ < 0.05) Tukey post-hoc tests within groups and between sessions were conducted.

Regarding the potential effects of individual changes in HF subfield volumes on clinical outcomes (3rd research question), we computed multiple linear regression models to examine the general associations between changes in HF subfield volume and changes in psychiatric symptoms, cognition, and functioning. Respective corresponding subfield volume differences between *t*0 and *t*6, group, age, sex, chlorpromazine equivalents, training number, study site, and years of education served as predictors. The difference in the corresponding behavioral scores between *t*0 and *t*6 was utilized as the dependent variable. *p* values of the volume differences were FDR-corrected.

## Results

### Search results, included studies and participant details

**Figure 1.**
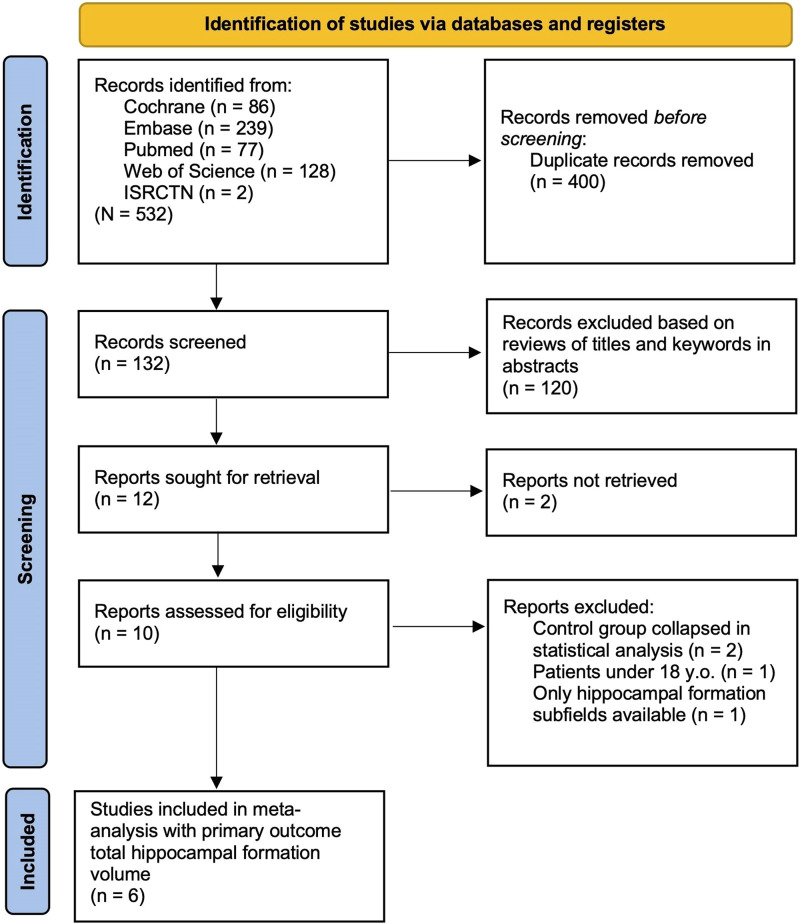
PRISMA flow diagram showcasing the process of identifying literature with total hippocampal formation volume as the primary outcome. A total of 6 studies were included in the meta-analysis.

As shown in Fig. 1, a total of 532 records were identified across five platforms. After removal of 400 duplicates, TF and LR screened 132 titles and abstracts, based on which an additional 120 studies were excluded. Of the remaining 12 records, 2 were not retrievable, 2 control conditions collapsed during primary analysis due to small sample sizes, 1 report included patients under the age of 18, and another only reported particular HF subfields. Thus, a total of six records were included in the primary analysis. The overall sample comprised 186 patients, 92 of which were assigned to aerobic exercise and 94 to control conditions. In total, 46% identified as female (*n* = 86) and 54% as male (*n* = 100), patients' mean age ranged from 24.6 to 39.3, and attrition rates from 0% to 79%. Studies were conducted in Germany (3x), Hong Kong, Iran, and the Netherlands (Khonsari et al., 2022; Lin et al., 2015; Malchow et al., 2016; Pajonk et al., 2010; Scheewe et al., 2013). Detailed patient and intervention study characteristics included in the meta-analyses can be found in online Supplementary Table S1. Risk of bias assessment suggested high concerns for three reports including our original data due to high attrition rates (Lin et al., 2015; Scheewe et al., 2013). Detailed results of the RoB analysis can be found in online Supplementary Figs S1 and S2.

#### Effect of aerobic exercise on global hippocampal formation volume assessed in meta-analysis

**Figure 2.**
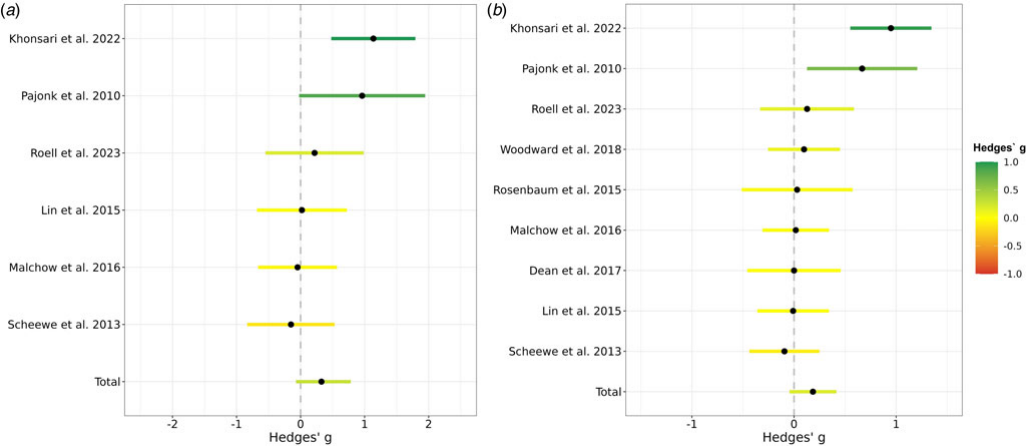
Effects of aerobic exercise on total hippocampal formation volume in people with schizophrenia. (a) Meta-analysis based on all trials with control condition comparing the effects on hippocampal formation volume in an aerobic exercise intervention to a study-specific control group. A positive Hedges g indicates that the volume increase in the aerobic exercise group was higher than in the control group. The 95% confidence intervals are represented by the colored lines. The pooled effect across all available studies is shown at the bottom and is labeled with ‘Total’. Roell et al. (2024) refers to the original data presented here. (b) Meta-analysis based on all available studies comparing the hippocampal formation volume prior to the aerobic exercise intervention with the volume after the aerobic exercise intervention. A positive Hedges' g indicates that there was a volume increase from baseline to the end of the intervention within the aerobic exercise group. The lines reflect the 95% confidence intervals. The pooled effect across all available studies is shown at the bottom and is labeled with ‘Total’. Roell et al. (2024) refers to the original data presented here.

As shown in Fig. 2a, the between-groups random-effects meta-analysis (*k* = 6) revealed a small to medium but non-significant effect of exercise interventions on global hippocampal formation volume (Hedges' *g* = 0.33, 95% CI −0.12 to 0.77, *p* = 0.15). Heterogeneity was substantial (*Q* = 11.52, *df* = 5, *p* = 0.04, *I^2^* = 56.58), whereas publication bias was non-significant (Kendall's tau = 0.27, *p* = 0.45). A funnel plot (online Supplementary Fig. S3), explored for asymmetry by means of Egger's Test, confirmed a lack of publication bias (Egger's intercept = 3.31, s.e. = 4.72, *p* = 0.52). Via removing one study at a time, the effect size of aerobic exercise on hippocampal volume ranged from 0.10 to 0.43.

As illustrated in Fig. 2b, the within-group random-effects meta-analysis (*k* = 9) revealed a small but non-significant effect of exercise interventions on global hippocampal formation volume in schizophrenia and related disorders (Hedges' *g* = 0.19, 95% CI −0.05 to 0.42, *p* = 0.11). Heterogeneity was substantial (*Q* = 23.08, *df* = 8, *p* < 0.003, *I^2^* = 65.34), whereas publication bias was non-significant (Kendall's tau = 0.42, *p* = 0.12). A funnel plot (online Supplementary Fig. S4), explored for asymmetry by means of Egger's Test, confirmed a lack of publication bias (Egger's intercept = 2.98, s.e. = 3.22, *p* = 0.39). Via removing one study at a time, the effect size of aerobic exercise on hippocampal volume ranged from 0.06 to 0.23. All test statistics are available in online Supplementary Table S3.

#### Effect of aerobic exercise on hippocampal formation subfield volumes assessed in RCT

**Figure 3.**
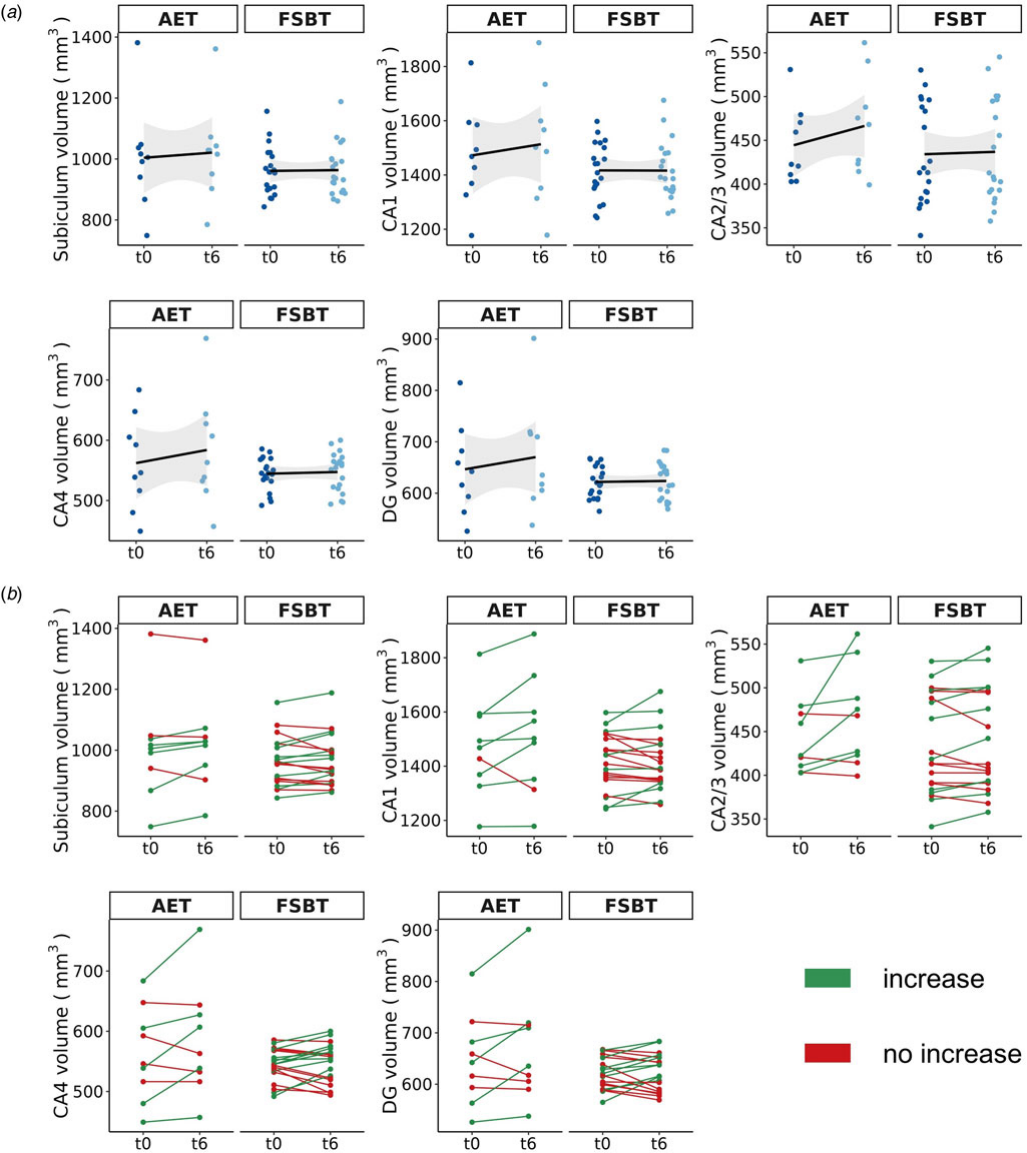
Exercise-induced volume changes of the HF subfields. AET, aerobic endurance training, FSBT, flexibility, strengthening and balance training; CA, cornu ammonis; DG, dentate gyrus; t0, baseline time point; t6, time point after six months of exercise. (a) The mean volume changes per subfield within each exercise group from time point t0 to t6 are shown. The shadowed area represents the 95% confidence interval. (b) The individual volume changes per subfield within each exercise group from time point *t*0 to *t*6 are displayed.

**Figure 4.**
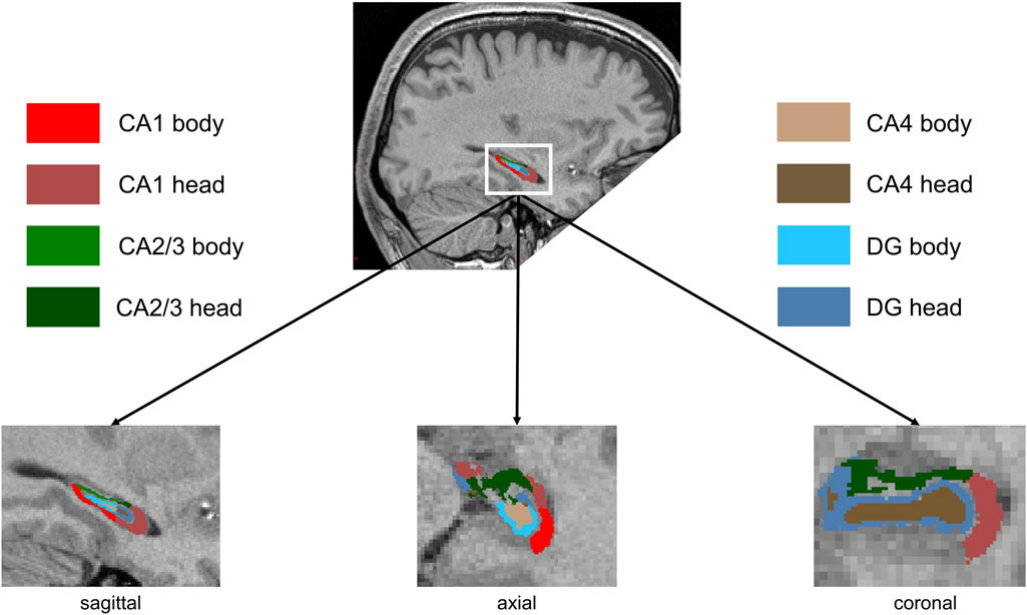
Hippocampal formation subfields. Sagittal (left), axial (middle) and coronal (right) view of the segmentation of CA1–4 and the DG. The subiculum is not displayed because no exercise effects were observed in this case. CA1–4 and DG are separated into head and body in this figure, but volumes from both parts were summed up for the statistical analysis. Images were acquired at a spatial isotropic resolution of 0.8 mm^3^ at study site Munich and 1 mm^3^ at study sites Mannheim and Berlin. CA, cornu ammonis; DG, dentate gyrus.

After FDR correction, we observed significant effects for the factor time and group × time interaction for the following subfields: CA1 (*F* = 5.11, *p*_FDR_ = 0.033), CA2/3 (*F* = 7.62, *p*_FDR_ = 0.033), CA4 (*F* = 5.29, *p*_FDR_ = 0.033), and DG (*F* = 5.67, *p*_FDR_ = 0.033). Thus, indicating significant differences in volume changes between AET and FSBT groups over time regarding all subfields except for the subiculum. Tukey post-hoc tests revealed significant volume increases between timepoints *t*0 and *t*6 for CA1 (*d* = 0.89, CI [0.22–1.55], *p*_tukey_ = 0.009), CA2/3 (*d* = 1.27, CI [0.61–1.93], *p*_tukey_ < 0.001), CA4 (*d* = 1.06, CI [0.40–1.73], *p*_tukey_ = 0.002), and DG (*d* = 1.04, CI [0.38–1.70], *p*_tukey_ = 0.002) in the AET, but not in the FSBT group. Figure 3 illustrates the effects on HF volumes in both exercise groups and Fig. 4 visualizes the subfields CA1–4 and the DG.

We did not observe any significant effects for the factor hemisphere (CA1: *p* = 0.864, CA2/3: *p* = 0.658, CA4: *p* = 0.335, DG: *p* = 0.392), suggesting that volume changes occur bilaterally to the same extent.

As part of our additional analysis, Tukey post-hoc tests indicated significant volume increases in the aerobic exercise group between timepoint *t*0 and *t*6 for the hippocampal head (*d* = 0.86, CI [0.20–1.53], *p*_tukey_ = 0.010) and body (*d* = 1.16, CI [0.50–1.82], *p*_tukey_ = 0.001), but not for the tail (*d* = 0.50, CI [−0.16 to 1.16], *p*_tukey_ = 0.137).

Clinical relevance of changes in HF volumes online Supplementary Table S4 and Figs S7, S8 and S9 summarize the full test statistics of the multiple linear regressions and the associations between changes in HF subfield volumes and changes in symptoms, cognition, and functioning, respectively. No stable correlations between longitudinal volumetric and psychopathological or cognitive changes were observed.

## Discussion

Our meta-analysis reveals that there was no significant effect of aerobic exercise on total HF volume. Rather, preliminary data from our randomized-controlled trial suggested that aerobic exercise may lead to significant volume increases in certain HF subfields, namely CA1, CA2/3, CA4, and DG. However, the clinical impact of said volume changes remains subject to further investigation.

Structural deteriorations within the HF play a key role in the pathophysiology of schizophrenia and contribute to cognitive deficits as well as positive and negative symptom severity (Adriano et al., 2012; Antoniades et al., 2018; Brosch et al., 2022; Brugger & Howes, 2017; Haukvik et al., 2018; Ho et al., 2017; Honea et al., 2005; Khalil et al., 2022; Nakahara et al., 2018, 2020; Opel et al., 2020; Pijnenborg et al., 2020; van Erp et al., 2016). Therefore, the implementation of interventions that promote hippocampal health constitute an essential approach towards improving treatment outcomes in schizophrenia. Although our meta-analysis did not demonstrate a significant effect of aerobic exercise on HF volume in people with schizophrenia, a noticeable trend in favor of aerobic exercise *v.* control conditions was observable. This is in line with previous meta-analyses conducted among various populations suggesting trends towards aerobic exercise-induced volume increases of the total HF (Firth et al., 2018; Li et al., 2017), but heterogeneity between studies is high. For instance, the included trials differ in several important properties such as the duration and the exact type of the utilized aerobic exercise programs, the kind of control group or the clinical status of the underlying study population. We systematically reviewed these properties, aiming at identifying a pattern that may explain why certain trials observe HF volume increases and others do not, but based on the available information no stable and systematic pattern was detectable. Hence, so far, we can only conclude that aerobic exercise interventions in people with schizophrenia may not consistently lead to HF volume increases on a group level, but rather affect HF volumes only under certain conditions:

Firstly, temporally restricted aerobic exercise interventions in the context of scientific studies may not be sufficient to observe stable HF volume increases. Instead, large-scale evidence demonstrates that an increased HF volume in healthy subjects is associated with a higher aerobic fitness level which in turn depends on the individual's longitudinal engagement in aerobic exercise (Wittfeld et al., 2020). Our previous cross-sectional findings in people with schizophrenia support this notion (Maurus et al., 2022a, 2022b), insofar as improvements of aerobic fitness during temporarily restricted aerobic exercise interventions have been linked to more pronounced increases of HF volume (Pajonk et al., 2010). Correspondingly, a recent meta-analysis comprising studies of various populations suggested that aerobic exercise interventions with a minimum duration of at least six months lead to more consistent increases in HF volume (Wilckens et al., 2021). Hence, we conclude that stable increases in HF volume as elicited by aerobic exercise are subject to long-term processes not always observable across the whole study population in the context of time-restricted intervention studies. Therefore, future cohort studies examining the long-term disease course of schizophrenia should collect measures of physical activity and fitness to better understand how frequently and over what period of time aerobic exercise should be performed to observe stable restorative effects on the HF volume across the whole study sample.

Second, however, aerobic exercise-induced HF volume increases can still be achieved within a shorter period of time (Khonsari et al., 2022; Pajonk et al., 2010), which may be attributable to the respective sample characteristics. Accordingly, previous evidence indicates that both a higher general polygenic risk for schizophrenia and a higher genetic burden associated with oligodendrocyte precursor cells and radial glia inhibit neuroplasticity within the HF during aerobic exercise (Papiol et al., 2017, 2019). These findings emphasize that certain genetic subgroups of patients are less likely to benefit from aerobic exercise interventions, which may in turn partially explain a lack of significance in observed effects on the group level. Hence, future research should identify multimodal factors that explain why certain patients with schizophrenia reveal increases in HF volume and others do not in order to enable individualized aerobic exercise treatments in future health care.

Thirdly, the potential effects of aerobic exercise on the HF may not be observable as a whole, but rather via subfields CA1, CA2/3, CA4 and DG, as indicated by our present preliminary study data. Accordingly, previous evidence indicates that different areas of the CA were particularly responsive to aerobic fitness and exercise (Maurus et al., 2022a; Woodward et al., 2018), whereas the subiculum, for example, was not (Damme et al., 2022). Moreover, there may also exist a hemisphere-specific effect of aerobic exercise on HF volume (Firth et al., 2018; Li et al., 2017). With regard to the underlying mechanisms, beneficial structural adjustments within the HF may result from several exercise-induced neural adaptations such as upregulations of neurotrophic factors, facilitated neuroplastic processes including neurogenesis, angiogenesis and gliogenesis or increases in dendritic density and length (Kandola, Hendrikse, Lucassen, & Yücel, 2016; Liu & Nusslock, 2018; Maurus et al., 2019). Additionally, ameliorations of physical health after aerobic exercise treatment (Vancampfort et al., 2015) may modulate neural plasticity in the HF subfields. Particularly, impaired physical and metabolic health plays a substantial role in schizophrenia (Tian et al., 2023) and thus can lead to widespread neural deteriorations in both brain structure and function (Herrmann, Tesar, Beier, Berg, & Warrings, 2019; Li et al., 2022; Parsons, Steward, Clohesy, Almgren, & Duehlmeyer, 2022; Syan et al., 2021). Improving physical and metabolic health through aerobic exercise treatments may thus yield the potential to stimulate regenerative processes in relevant brain regions such as the HF. Considering that CA subfields are especially prone to volume loss in schizophrenia (Ho et al., 2017; Park et al., 2021), it remains particularly important to emphasize that exercise-induced volume increases are assumed to be restorative in their nature, thereby counteracting age- and/or disorder-dependent volume decline (Firth et al., 2018; Li et al., 2017). However, as indicated by our data, the group effect of volume increases in the CA and DG is mainly driven by a few subjects in the aerobic exercise group that show particularly pronounced volume increases (Fig. 3b). This suggests that there is a subgroup of patients with schizophrenia that is specifically responsive to aerobic exercise, potentially modulated by lower general polygenic risk for schizophrenia and a higher genetic burden associated with oligodendrocyte precursor cells and radial glia (Papiol et al., 2017, 2019). Importantly, more large-scale exercise trials in people with schizophrenia are required to identify stable subgroups of patients that consistently show increased neuroplasticity in the CA and DG after aerobic exercise. To conclude, aerobic exercise potentially mitigates neurodegeneration of one of the most affected HF subfields in people with schizophrenia, at least for a subgroup of patients.

Structural decline of the HF and its subfields is associated with severe cognitive impairments and schizophrenic symptoms (Antoniades et al., 2018; Haukvik et al., 2018; Ho et al., 2017; Khalil et al., 2022; Nakahara et al., 2018, 2020; Pijnenborg et al., 2020). Persistent cognitive deficits prevent long-term improvements in the patients' social and occupational functioning and thus contribute to an unfavorable disease outcome (Green, 2016). In this regard, current large-scale evidence appears promising, demonstrating that exercise treatments improve both cognitive functioning (Dauwan et al., 2016; Fernández-Abascal et al., 2021; Korman et al., 2023) and daily life functioning (Dauwan et al., 2016; Fernández-Abascal et al., 2021; Korman et al., 2023) in schizophrenia. Given the aforementioned link between structural decline within the HF and clinical and cognitive symptoms in schizophrenia, we expected individual-specific HF volume changes post exercise intervention to be linked to changes in clinical outcomes. However, we did not observe said effects, thereby contradicting previous studies suggesting volume increases in the HF to be linked to improvements in short-term memory and global functioning (Falkai et al., 2021; Pajonk et al., 2010). As corresponding evidence in people with schizophrenia is still limited, we cannot yet derive definite conclusions regarding the clinical relevance of exercise-induced structural adaptations of the HF. Because the stable detection of brain-behavior associations in general requires well-powered large-scale studies, those are needed to clarify the clinical implications of aerobic exercise-induced increases in HF subfield volumes in people with schizophrenia. Thereby, future trials should include comprehensive HF-related cognitive test batteries, covering domains such as episodic or visuospatial memory.

Limitations of this study include that the conclusions regarding the HF subfields are preliminary and require further replication in a larger independent sample. As the MRI assessments were not part of the primary endpoint of the ESPRIT C3 study (for details see Maurus et al. (2020) and Maurus et al. (2023)) and thus were not financially compensated, only a subgroup of patients agreed to participate. Consequently, future studies should ensure adequate financial compensation for patients undergoing MRI assessments since reservations and fears regarding this method are particularly pronounced in schizophrenia. Furthermore, the FreeSurfer segmentation strategy for the HF subfields based on MRI scans at a spatial isotropic resolution of 1 mm^3^, as applied at study sites Mannheim and Berlin, has been criticized due to issues regarding the detection of the boundaries between subfields (Wisse et al., 2021). However, we still observe distinct patterns of volume increases within the HF when subdividing it only into head, body, and tail, which is less prone to bias. Moreover, the current data basis was not sufficient to identify subgroups of people with schizophrenia who show pronounced volume increases after aerobic exercise. Lastly, methodological differences across the considered exercise studies in schizophrenia complicate a reasonable quantitative approach to summarize findings regarding the clinical relevance of potential HF volume changes in response to aerobic exercise.

In sum, we conclude that aerobic exercise interventions in people with schizophrenia do not lead to significant increases in the total HF volume on the population level. However, our study provides preliminary evidence that aerobic exercise may lead to increases in specific subfields of the HF, namely CA1, CA2/3, CA4, and DG. Large-scale aerobic exercise studies in people with schizophrenia are required to further investigate said effects of aerobic exercise on HF subfield volumes, to identify subgroups of patients that respond particularly well to aerobic exercise, and to elucidate the clinical implications of volume changes within the HF.

## Supporting information

Roell et al. supplementary material Roell et al. supplementary material

## Data Availability

The data that support the findings of this study are available on request from the corresponding author, LR. The data are not publicly available because it contains information that could compromise the privacy of research participants. All analysis scripts and documentation sheets will be published on OSF (DOI 10.17605/OSF.IO/TR3NX). A preprint is available under https://doi.org/10.31219/osf.io/y2phs.

## Supplementary material

The supplementary material for this article can be found at https://doi.org/10.1017/S0033291724001867

## Clinical trials registration

The underlying study of this manuscript was registered in the International Clinical Trials Database, ClinicalTrials.gov (NCT number: NCT03466112, https://clinicaltrials.gov/ct2/show/NCT03466112?term=NCT03466112&draw=2&rank=1) and in the German Clinical Trials Register (DRKS-ID: DRKS00009804).

## References

[ref1] Adriano, F., Caltagirone, C., & Spalletta, G. (2012). Hippocampal volume reduction in first-episode and chronic schizophrenia: A review and meta-analysis. The Neuroscientist, 18(2), 180–200. doi: 10.1177/1073858410395147 21531988

[ref2] Antoniades, M., Schoeler, T., Radua, J., Valli, I., Allen, P., Kempton, M. J., & McGuire, P. (2018). Verbal learning and hippocampal dysfunction in schizophrenia: A meta-analysis. Neuroscience & Biobehavioral Reviews, 86, 166–175. doi: 10.1016/j.neubiorev.2017.12.001 29223768 PMC5818020

[ref3] Begg, C. B., & Mazumdar, M. (1994). Operating characteristics of a rank correlation test for publication bias. Biometrics, 50(4), 1088–1101. 7786990

[ref4] Benjamini, Y., & Hochberg, Y. (1995). Controlling the false discovery rate: A practical and powerful approach to multiple testing. Journal of the Royal Statistical Society, 57(1), 289–300. doi: 10.2307/2346101

[ref5] Bird, C. M., & Burgess, N. (2008). The hippocampus and memory: Insights from spatial processing. Nat Reviews in the Neurosciences, 9(3), 182–194. doi: 10.1038/nrn2335 18270514

[ref6] Borenstein, M. (2022). Comprehensive meta-analysis software. In M. Egger, J. P. T. Higgins, & G. D. Smith (Eds.), Systematic reviews in health research (pp. 535–548). New Jersey: Wiley.

[ref7] Brosch, K., Stein, F., Schmitt, S., Pfarr, J. K., Ringwald, K. G., Thomas-Odenthal, F., … Kircher, T. (2022). Reduced hippocampal gray matter volume is a common feature of patients with major depression, bipolar disorder, and schizophrenia spectrum disorders. Molecular Psychiatry, 27(10), 4234–4243. doi: 10.1038/s41380-022-01687-4 35840798 PMC9718668

[ref8] Brugger, S. P., & Howes, O. D. (2017). Heterogeneity and homogeneity of regional brain structure in schizophrenia: A meta-analysis. JAMA Psychiatry, 74(11), 1104–1111. doi: 10.1001/jamapsychiatry.2017.2663 28973084 PMC5669456

[ref9] Chopra, S., Segal, A., Oldham, S., Holmes, A., Sabaroedin, K., Orchard, E. R., … Fornito, A. (2023). Network-based spreading of gray matter changes across different stages of psychosis. JAMA Psychiatry, 80(12), 1246–1257. doi: 10.1001/jamapsychiatry.2023.3293. 37728918 PMC10512169

[ref10] Damme, K. S. F., Gupta, T., Ristanovic, I., Kimhy, D., Bryan, A. D., & Mittal, V. A. (2022). Exercise intervention in individuals at clinical high risk for psychosis: Benefits to fitness, symptoms, hippocampal volumes, and functional connectivity. Schizophrenia Bulletin, 48(6), 1394–1405. doi: 10.1093/schbul/sbac084 35810336 PMC9673264

[ref11] Dauwan, M., Begemann, M. J., Heringa, S. M., & Sommer, I. E. (2016). Exercise improves clinical symptoms, quality of life, global functioning, and depression in schizophrenia: A systematic review and meta-analysis. Schizoprenia Bulletin, 42(3), 588–599. doi: 10.1093/schbul/sbv164 PMC4838091 26547223

[ref12] Dean, D. J., Bryan, A. D., Newberry, R., Gupta, T., Carol, E., & Mittal, V. A. (2017). A supervised exercise intervention for youth at risk for psychosis: An open-label pilot study. Journal of Clinical Psychiatry, 78(9), 1167–1173. doi: 10.4088/JCP.16m11365 PMC5995728 29178684

[ref13] Duncan, K., Ketz, N., Inati, S. J., & Davachi, L. (2012). Evidence for area CA1 as a match/mismatch detector: A high-resolution fMRI study of the human hippocampus. Hippocampus, 22(3), 389–398. doi: 10.1002/hipo.20933 21484934 PMC3529001

[ref14] Duval, S., & Tweedie, R. (2000). Trim and fill: A simple funnel-plot-based method of testing and adjusting for publication bias in meta-analysis. Biometrics, 56(2), 455–463. doi: 10.1111/j.0006-341x.2000.00455.x 10877304

[ref15] Egger, M., Davey Smith, G., Schneider, M., & Minder, C. (1997). Bias in meta-analysis detected by a simple, graphical test. BMJ, 315(7109), 629–634. doi: 10.1136/bmj.315.7109.629 9310563 PMC2127453

[ref16] Eldridge, L. L., Engel, S. A., Zeineh, M. M., Bookheimer, S. Y., & Knowlton, B. J. (2005). A dissociation of encoding and retrieval processes in the human hippocampus. Journal of Neuroscience, 25(13), 3280–3286. doi: 10.1523/jneurosci.3420-04.2005 15800182 PMC6724896

[ref17] Endicott, J., Spitzer, R. L., Fleiss, J. L., & Cohen, J. (1976). The global assessment scale. A procedure for measuring overall severity of psychiatric disturbance. Archives of General Psychiatry, 33(6), 766–771. doi: 10.1001/archpsyc.1976.01770060086012 938196

[ref18] Falkai, P., Steiner, J., Malchow, B., Shariati, J., Knaus, A., Bernstein, H. G., … Schmitt, A. (2016). Oligodendrocyte and interneuron density in hippocampal subfields in schizophrenia and association of oligodendrocyte number with cognitive deficits. Frontiers in Celulularl Neuroscience, 10, 78. doi: 10.3389/fncel.2016.00078 PMC4811909 27065804

[ref19] Falkai, P., Maurus, I., Schmitt, A., Malchow, B., Schneider-Axmann, T., Röll, L., … Keeser, D. (2021). Improvement in daily functioning after aerobic exercise training in schizophrenia is sustained after exercise cessation. European Archives of Psychiatry and Clinical Neuroscience, 271(7), 1201–1203. doi: 10.1007/s00406-021-01282-8 34143287 PMC8429390

[ref20] Fernández-Abascal, B., Suárez-Pinilla, P., Cobo-Corrales, C., Crespo-Facorro, B., & Suárez-Pinilla, M. (2021). In- and outpatient lifestyle interventions on diet and exercise and their effect on physical and psychological health: A systematic review and meta-analysis of randomised controlled trials in patients with schizophrenia spectrum disorders and first episode of psychosis. Neuroscience & Biobehavioral Reviews, 125, 535–568. doi: 10.1016/j.neubiorev.2021.01.005 33503476

[ref21] Firth, J., Cotter, J., Elliott, R., French, P., & Yung, A. R. (2015). A systematic review and meta-analysis of exercise interventions in schizophrenia patients. Psychological Medicine, 45(7), 1343–1361. doi: 10.1017/s0033291714003110 25650668

[ref22] Firth, J., Cotter, J., Carney, R., & Yung, A. R. (2017a). The pro-cognitive mechanisms of physical exercise in people with schizophrenia. British Journal of Pharmacology, 174(19), 3161–3172. doi: 10.1111/bph.13772 28261797 PMC5595765

[ref23] Firth, J., Stubbs, B., Rosenbaum, S., Vancampfort, D., Malchow, B., Schuch, F., … Yung, A. R. (2017b). Aerobic exercise improves cognitive functioning in people with schizophrenia: A systematic review and meta-analysis. Schizoprenia Bulletin, 43(3), 546–556. doi: 10.1093/schbul/sbw115 PMC5464163 27521348

[ref24] Firth, J., Stubbs, B., Vancampfort, D., Schuch, F., Lagopoulos, J., Rosenbaum, S., & Ward, P. B. (2018). Effect of aerobic exercise on hippocampal volume in humans: A systematic review and meta-analysis. Neuroimage, 166, 230–238. doi: 10.1016/j.neuroimage.2017.11.007 29113943

[ref25] Gallardo-Gómez, D., Noetel, M., Álvarez-Barbosa, F., Alfonso-Rosa, R. M., Ramos-Munell, J., Del Pozo Cruz, B., & Del Pozo-Cruz, J. (2023). Exercise to treat psychopathology and other clinical outcomes in schizophrenia: A systematic review and meta-analysis. European Psychiatry, 66(1), e40. doi: 10.1192/j.eurpsy.2023.24 37096668 PMC10305321

[ref26] Green, M. F. (2016). Impact of cognitive and social cognitive impairment on functional outcomes in patients with schizophrenia. Journal of Clinical Psychiatry, 77(Suppl 2), 8–11. doi: 10.4088/JCP.14074su1c.02 26919052

[ref27] Guo, J., Liu, K., Liao, Y., Qin, Y., & Yue, W. (2024). Efficacy and feasibility of aerobic exercise interventions as an adjunctive treatment for patients with schizophrenia: A meta-analysis. Schizophrenia, 10(1), 2. doi: 10.1038/s41537-023-00426-0 38167923 PMC10851701

[ref28] Hainmueller, T., & Bartos, M. (2020). Dentate gyrus circuits for encoding, retrieval and discrimination of episodic memories. Nature Reviews Neuroscience, 21(3), 153–168. doi: 10.1038/s41583-019-0260-z 32042144 PMC7115869

[ref29] Haukvik, U. K., Tamnes, C. K., Söderman, E., & Agartz, I. (2018). Neuroimaging hippocampal subfields in schizophrenia and bipolar disorder: A systematic review and meta-analysis. Journal of Psychiatric Research, 104, 217–226. doi: 10.1016/j.jpsychires.2018.08.012 30107268

[ref30] Helmstaedter, C., & Durwen, H. F. (1990). VLMT: A useful tool to assess and differentiate verbal memory performance. Swiss Archives of Neurology, Psychiatry and Psychotherapy, 141, 21–30. 1690447

[ref31] Herrmann, M. J., Tesar, A. K., Beier, J., Berg, M., & Warrings, B. (2019). Grey matter alterations in obesity: A meta-analysis of whole-brain studies. Obesity Reviews, 20(3), 464–471. doi: 10.1111/obr.12799 30537231

[ref32] Ho, N. F., Iglesias, J. E., Sum, M. Y., Kuswanto, C. N., Sitoh, Y. Y., De Souza, J., … Holt, D. J. (2017). Progression from selective to general involvement of hippocampal subfields in schizophrenia. Molecular Psychiatry, 22(1), 142–152. doi: 10.1038/mp.2016.4 26903271 PMC4995163

[ref33] Honea, R., Crow, T. J., Passingham, D., & Mackay, C. E. (2005). Regional deficits in brain volume in schizophrenia: A meta-analysis of voxel-based morphometry studies. American Journal of Psychiatry, 162(12), 2233–2245. doi: 10.1176/appi.ajp.162.12.2233 16330585

[ref34] Iglesias, J. E., Augustinack, J. C., Nguyen, K., Player, C. M., Player, A., Wright, M., … Van Leemput, K. (2015). A computational atlas of the hippocampal formation using ex vivo, ultra-high resolution MRI: Application to adaptive segmentation of in vivo MRI. Neuroimage, 115, 117–137. doi: 10.1016/j.neuroimage.2015.04.042 25936807 PMC4461537

[ref35] Kandola, A., Hendrikse, J., Lucassen, P. J., & Yücel, M. (2016). Aerobic exercise as a tool to improve hippocampal plasticity and function in humans: Practical implications for mental health treatment. Frontiers in Human Neuroscience, 10, 373. doi: 10.3389/fnhum.2016.00373 27524962 PMC4965462

[ref36] Kay, S. R., Fiszbein, A., & Opler, L. A. (1987). The positive and negative syndrome scale (PANSS) for schizophrenia. Schizoprenia Bulletin, 13(2), 261–276. doi: 10.1093/schbul/13.2.261 3616518

[ref37] Khalil, M., Hollander, P., Raucher-Chéné, D., Lepage, M., & Lavigne, K. M. (2022). Structural brain correlates of cognitive function in schizophrenia: A meta-analysis. Neuroscience & Biobehavioral Reviews, 132, 37–49. doi: 10.1016/j.neubiorev.2021.11.034 34822878

[ref38] Khonsari, N. M., Badrfam, R., Mohammdi, M. R., Rastad, H., Etemadi, F., Vafaei, Z., & Zandifar, A. (2022). Effect of aerobic exercise as adjunct therapy on the improvement of negative symptoms and cognitive impairment in patients with schizophrenia: A randomized, case-control clinical trial. Journal of Psychosocial Nursing and Mental Health Services, 60(5), 38–43. doi: 10.3928/02793695-20211014-03 34677118

[ref39] Kim, M., Lee, Y., & Kang, H. (2023). Effects of exercise on positive symptoms, negative symptoms, and depression in patients with schizophrenia: A systematic review and meta-analysis. International Journal of Environmental Research and Public Health, 20(4), 3719. doi: 10.3390/ijerph20043719 36834415 PMC9967614

[ref40] Korman, N., Stanton, R., Vecchio, A., Chapman, J., Parker, S., Martland, R., … Firth, J. (2023). The effect of exercise on global, social, daily living and occupational functioning in people living with schizophrenia: A systematic review and meta-analysis. Schizophrenia Research, 256, 98–111. doi: 10.1016/j.schres.2023.04.012 37209456

[ref41] Li, M. Y., Huang, M. M., Li, S. Z., Tao, J., Zheng, G. H., & Chen, L. D. (2017). The effects of aerobic exercise on the structure and function of DMN-related brain regions: A systematic review. International Journal of Neuroscience, 127(7), 634–649. doi: 10.1080/00207454.2016.1212855 27412353

[ref42] Li, L., Yu, H., Zhong, M., Liu, S., Wei, W., Meng, Y., … Wang, Q. (2022). Gray matter volume alterations in subjects with overweight and obesity: Evidence from a voxel-based meta-analysis. Frontiers in Psychiatry, 13, 955741. doi: 10.3389/fpsyt.2022.955741 36226110 PMC9548618

[ref43] Lin, J., Chan, S. K., Lee, E. H., Chang, W. C., Tse, M., Su, W. W., … Chen, E. Y. (2015). Aerobic exercise and yoga improve neurocognitive function in women with early psychosis. NPJ Schizophrenia, 1(0), 15047. doi: 10.1038/npjschz.2015.47 27336050 PMC4849465

[ref44] Liu-Ambrose, T., Nagamatsu, L. S., Graf, P., Beattie, B. L., Ashe, M. C., & Handy, T. C. (2010). Resistance training and executive functions: A 12-month randomized controlled trial. Archives of International Medicine, 170(2), 170–178. doi: 10.1001/archinternmed.2009.494 PMC3448565 20101012

[ref45] Liu, P. Z., & Nusslock, R. (2018). Exercise-mediated neurogenesis in the hippocampus via BDNF. Frontiers in Neuroscience, 12, 52. doi: 10.3389/fnins.2018.00052 29467613 PMC5808288

[ref46] Llorca, P. M., Lançon, C., Lancrenon, S., Bayle, F. J., Caci, H., Rouillon, F., & Gorwood, P. (2009). The “Functional Remission of General Schizophrenia” (FROGS) scale: Development and validation of a new questionnaire. Schizophrenia Research, 113(2-3), 218–225. doi: 10.1016/j.schres.2009.04.029 19464855

[ref47] Malchow, B., Keeser, D., Keller, K., Hasan, A., Rauchmann, B. S., Kimura, H., … Falkai, P. (2016). Effects of endurance training on brain structures in chronic schizophrenia patients and healthy controls. Schizophrenia Research, 173(3), 182–191. doi: 10.1016/j.schres.2015.01.005 25623601

[ref48] Mathew, I., Gardin, T. M., Tandon, N., Eack, S., Francis, A. N., Seidman, L. J., … Keshavan, M. S. (2014). Medial temporal lobe structures and hippocampal subfields in psychotic disorders: Findings from the Bipolar-Schizophrenia Network on Intermediate Phenotypes (B-SNIP) study. JAMA Psychiatry, 71(7), 769–777. doi: 10.1001/jamapsychiatry.2014.453 24828364

[ref49] Maurus, I., Hasan, A., Röh, A., Takahashi, S., Rauchmann, B., Keeser, D., … Falkai, P. (2019). Neurobiological effects of aerobic exercise, with a focus on patients with schizophrenia. European Archives of Psychiatry and Clinical Neuroscience, 269(5), 499–515. doi: 10.1007/s00406-019-01025-w 31115660

[ref50] Maurus, I., Hasan, A., Schmitt, A., Roeh, A., Keeser, D., Malchow, B., … Falkai, P. (2020). Aerobic endurance training to improve cognition and enhance recovery in schizophrenia: Design and methodology of a multicenter randomized controlled trial. European Archives of Psychiatry and Clinical Neuroscience, 271, 315–324. doi: 10.1007/s00406-020-01175-2 32748261 PMC8257533

[ref51] Maurus, I., Roell, L., Keeser, D., Papazov, B., Papazova, I., Lembeck, M., … Falkai, P. (2022a). Fitness is positively associated with hippocampal formation subfield volumes in schizophrenia: A multiparametric magnetic resonance imaging study. Translational Psychiatry, 12(1), 388. doi: 10.1038/s41398-022-02155-x 36114184 PMC9481539

[ref52] Maurus, I., Röll, L., Keeser, D., Karali, T., Papazov, B., Hasan, A., … Falkai, P. (2022b). Associations between aerobic fitness, negative symptoms, cognitive deficits and brain structure in schizophrenia-a cross-sectional study. Schizophrenia, 8(1), 63. doi: 10.1038/s41537-022-00269-1 35918344 PMC9345912

[ref53] Maurus, I., Roell, L., Lembeck, M., Papazova, I., Greska, D., Muenz, S., … Falkai, P. (2023). Exercise as an add-on treatment in individuals with schizophrenia: Results from a large multicenter randomized controlled trial. Psychiatry Research, 328, 115480. doi: 10.1016/j.psychres.2023.115480 37716320

[ref54] Nakahara, S., Matsumoto, M., & van Erp, T. G. M. (2018). Hippocampal subregion abnormalities in schizophrenia: A systematic review of structural and physiological imaging studies. Neuropsychopharmacology Reports, 38(4), 156–166. doi: 10.1002/npr2.12031 30255629 PMC7021222

[ref55] Nakahara, S., Turner, J. A., Calhoun, V. D., Lim, K. O., Mueller, B., Bustillo, J. R., … van Erp, T. G. M. (2020). Dentate gyrus volume deficit in schizophrenia. Psycholological Medicine, 50(8), 1267–1277. doi: 10.1017/s0033291719001144 PMC7068799 31155012

[ref56] O'Brien, L. M., Ziegler, D. A., Deutsch, C. K., Frazier, J. A., Herbert, M. R., & Locascio, J. J. (2011). Statistical adjustments for brain size in volumetric neuroimaging studies: Some practical implications in methods. Psychiatry Research, 193(2), 113–122. doi: 10.1016/j.pscychresns.2011.01.007 21684724 PMC3510982

[ref57] Opel, N., Goltermann, J., Hermesdorf, M., Berger, K., Baune, B. T., & Dannlowski, U. (2020). Cross-Disorder analysis of brain structural abnormalities in six major psychiatric disorders: A secondary analysis of mega- and meta-analytical findings from the ENIGMA consortium. Biological Psychiatry, 88(9), 678–686. doi: 10.1016/j.biopsych.2020.04.027 32646651

[ref58] Ouzzani, M., Hammady, H., Fedorowicz, Z., & Elmagarmid, A. (2016). Rayyan – a web and mobile app for systematic reviews. Systematic Reviews, 5(1), 210. doi: 10.1186/s13643-016-0384-4 27919275 PMC5139140

[ref59] Pajonk, F. G., Wobrock, T., Gruber, O., Scherk, H., Berner, D., Kaizl, I., … Falkai, P. (2010). Hippocampal plasticity in response to exercise in schizophrenia. Archives of General Psychiatry, 67(2), 133–143. doi: 10.1001/archgenpsychiatry.2009.193 20124113

[ref60] Papiol, S., Popovic, D., Keeser, D., Hasan, A., Schneider-Axmann, T., Degenhardt, F., … Malchow, B. (2017). Polygenic risk has an impact on the structural plasticity of hippocampal subfields during aerobic exercise combined with cognitive remediation in multi-episode schizophrenia. Translational Psychiatry, 7(6), e1159. doi: 10.1038/tp.2017.131 28654095 PMC5537649

[ref61] Papiol, S., Keeser, D., Hasan, A., Schneider-Axmann, T., Raabe, F., Degenhardt, F., … Falkai, P. (2019). Polygenic burden associated to oligodendrocyte precursor cells and radial glia influences the hippocampal volume changes induced by aerobic exercise in schizophrenia patients. Translational Psychiatry, 9(1), 284. doi: 10.1038/s41398-019-0618-z 31712617 PMC6848123

[ref62] Park, M. T. M., Jeon, P., Khan, A. R., Dempster, K., Chakravarty, M. M., Lerch, J. P., … Palaniyappan, L. (2021). Hippocampal neuroanatomy in first episode psychosis: A putative role for glutamate and serotonin receptors. Progress in Neuro-Psychopharmacololgy and Biological Psychiatry, 110, 110297. doi: 10.1016/j.pnpbp.2021.110297 33691200

[ref63] Parsons, N., Steward, T., Clohesy, R., Almgren, H., & Duehlmeyer, L. (2022). A systematic review of resting-state functional connectivity in obesity: Refining current neurobiological frameworks and methodological considerations moving forward. Reviews in Endocrine and Metabolic Disorders, 23(4), 861–879. doi: 10.1007/s11154-021-09665-x 34159504

[ref64] Pijnenborg, G. H. M., Larabi, D. I., Xu, P., Hasson-Ohayon, I., de Vos, A. E., Ćurčić-Blake, B., … Van der Meer, L. (2020). Brain areas associated with clinical and cognitive insight in psychotic disorders: A systematic review and meta-analysis. Neuroscience & Biobehavioral Reviews, 116, 301–336. doi: 10.1016/j.neubiorev.2020.06.022 32569706

[ref65] R Core Team. (2021). R: A language and environment for statistical computing. Vienna, Austria: R Foundation for Statistical Computing. Retrieved from https://www.R-project.org/

[ref66] Roeske, M. J., Konradi, C., Heckers, S., & Lewis, A. S. (2021). Hippocampal volume and hippocampal neuron density, number and size in schizophrenia: A systematic review and meta-analysis of postmortem studies. Molecular Psychiatry, 26(7), 3524–3535. doi: 10.1038/s41380-020-0853-y 32724199 PMC7854798

[ref67] Rosenbaum, S., Lagopoulos, J., Curtis, J., Taylor, L., Watkins, A., Barry, B. K., & Ward, P. B. (2015). Aerobic exercise intervention in young people with schizophrenia spectrum disorders; improved fitness with no change in hippocampal volume. Psychiatry Research, 232(2), 200–201. doi: 10.1016/j.pscychresns.2015.02.004 25862528

[ref68] RStudio Team. (2020). RStudio: Integrated development environment for R. Boston, MA: RStudio, PBC. Retrieved from http://www.rstudio.com/

[ref69] Sabe, M., Kaiser, S., & Sentissi, O. (2020). Physical exercise for negative symptoms of schizophrenia: Systematic review of randomized controlled trials and meta-analysis. General Hospital Psychiatry, 62, 13–20. doi: 10.1016/j.genhosppsych.2019.11.002 31751931

[ref70] Scheewe, T. W., van Haren, N. E., Sarkisyan, G., Schnack, H. G., Brouwer, R. M., de Glint, M., … Cahn, W. (2013). Exercise therapy, cardiorespiratory fitness and their effect on brain volumes: A randomised controlled trial in patients with schizophrenia and healthy controls. European Neuropsychopharmacololgy, 23(7), 675–685. doi: 10.1016/j.euroneuro.2012.08.008 22981376

[ref71] Schmitt, A., Reich-Erkelenz, D., Hasan, A., & Falkai, P. (2019). Aerobic exercise in mental disorders: From basic mechanisms to treatment recommendations. European Archives of Psychiatry and Clinical Neuroscience, 269(5), 483–484. doi: 10.1007/s00406-019-01037-6 31250087

[ref72] Schmitt, A., Tatsch, L., Vollhardt, A., Schneider-Axmann, T., Raabe, F. J., Roell, L., … Schmitz, C. (2022). Decreased oligodendrocyte number in hippocampal subfield CA4 in schizophrenia: A replication study. Cells, 11(20), 3242. doi: 10.3390/cells11203242 36291109 PMC9600243

[ref73] Schultz, C., & Engelhardt, M. (2014). Anatomy of the hippocampal formation. Frontiers of Neurology and Neuroscience, 34, 6–17. doi: 10.1159/000360925 24777126

[ref74] Shimada, T., Ito, S., Makabe, A., Yamanushi, A., Takenaka, A., Kawano, K., & Kobayashi, M. (2022). Aerobic exercise and cognitive functioning in schizophrenia: An updated systematic review and meta-analysis. Psychiatry Research, 314, 114656. doi: 10.1016/j.psychres.2022.114656 35659670

[ref75] Sterne, J. A. C., Savović, J., Page, M. J., Elbers, R. G., Blencowe, N. S., Boutron, I., … Higgins, J. P. T. (2019). Rob 2: A revised tool for assessing risk of bias in randomised trials. BMJ, 366, l4898. doi: 10.1136/bmj.l4898 31462531

[ref76] Syan, S. K., McIntyre-Wood, C., Minuzzi, L., Hall, G., McCabe, R. E., & MacKillop, J. (2021). Dysregulated resting state functional connectivity and obesity: A systematic review. Neuroscience & Biobehavioral Reviews, 131, 270–292. doi: 10.1016/j.neubiorev.2021.08.019 34425125

[ref77] Tewes, U. (1994). Hamburg-Wechsler intelligenztest für erwachsene revision 1991 *(*HAWIE-R*)* (Vol. 2). Bern: Huber.

[ref78] Tian, Y. E., Di Biase, M. A., Mosley, P. E., Lupton, M. K., Xia, Y., Fripp, J., … Zalesky, A. (2023). Evaluation of brain-body health in individuals with common neuropsychiatric disorders. JAMA Psychiatry, 80(6), 567–576. doi: 10.1001/jamapsychiatry.2023.0791 37099313 PMC10134046

[ref79] Vancampfort, D., Rosenbaum, S., Ward, P. B., & Stubbs, B. (2015). Exercise improves cardiorespiratory fitness in people with schizophrenia: A systematic review and meta-analysis. Schizophrenia Research, 169(1-3), 453–457. doi: 10.1016/j.schres.2015.09.029 26475214

[ref80] van Erp, T. G., Hibar, D. P., Rasmussen, J. M., Glahn, D. C., Pearlson, G. D., Andreassen, O. A., … Turner, J. A. (2016). Subcortical brain volume abnormalities in 2028 individuals with schizophrenia and 2540 healthy controls via the ENIGMA consortium. Molecular Psychiatry, 21(4), 547–553. doi: 10.1038/mp.2015.63 26033243 PMC4668237

[ref81] Vogel, J. S., van der Gaag, M., Slofstra, C., Knegtering, H., Bruins, J., & Castelein, S. (2019). The effect of mind-body and aerobic exercise on negative symptoms in schizophrenia: A meta-analysis. Psychiatry Research, 279, 295–305. doi: 10.1016/j.psychres.2019.03.012 30879703

[ref82] Wei, G. X., Yang, L., Imm, K., Loprinzi, P. D., Smith, L., Zhang, X., & Yu, Q. (2020). Effects of mind-body exercises on schizophrenia: A systematic review with meta-analysis. Frontiers in Psychiatry, 11, 819. doi: 10.3389/fpsyt.2020.00819 32922321 PMC7457019

[ref83] Wickham, H. (2016). Ggplot2: Elegant graphics for data analysis. New York: Springer. Retrieved from https://ggplot2.tidyverse.org

[ref84] Wilckens, K. A., Stillman, C. M., Waiwood, A. M., Kang, C., Leckie, R. L., Peven, J. C., … Erickson, K. I. (2021). Exercise interventions preserve hippocampal volume: A meta-analysis. Hippocampus, 31(3), 335–347. doi: 10.1002/hipo.23292 33315276 PMC11497212

[ref85] Wisse, L. E. M., Chételat, G., Daugherty, A. M., de Flores, R., la Joie, R., Mueller, S. G., … Carr, V. A. (2021). Hippocampal subfield volumetry from structural isotropic 1 mm(3) MRI scans: A note of caution. Human Brain Mapping, 42(2), 539–550. doi: 10.1002/hbm.25234 33058385 PMC7775994

[ref86] Wittfeld, K., Jochem, C., Dörr, M., Schminke, U., Gläser, S., Bahls, M., … Grabe, H. J. (2020). Cardiorespiratory fitness and gray matter volume in the temporal, frontal, and cerebellar regions in the general population. Mayo Clinic Proceedings, 95(1), 44–56. doi: 10.1016/j.mayocp.2019.05.030 31902428

[ref87] Woodward, M. L., Gicas, K. M., Warburton, D. E., White, R. F., Rauscher, A., Leonova, O., … Lang, D. J. (2018). Hippocampal volume and vasculature before and after exercise in treatment-resistant schizophrenia. Schizophrenia Research, 202, 158–165. doi: 10.1016/j.schres.2018.06.054 30539767

[ref88] Xu, Y., Cai, Z., Fang, C., Zheng, J., Shan, J., & Yang, Y. (2022). Impact of aerobic exercise on cognitive function in patients with schizophrenia during daily care: A meta-analysis. Psychiatry Research, 312, 114560. doi: 10.1016/j.psychres.2022.114560 35500333

[ref89] Zeineh, M. M., Engel, S. A., Thompson, P. M., & Bookheimer, S. Y. (2003). Dynamics of the hippocampus during encoding and retrieval of face-name pairs. Science (New York, N.Y.), 299(5606), 577–580. doi: 10.1126/science.1077775 12543980

[ref90] Ziebart, C., Bobos, P., MacDermid, J. C., Furtado, R., Sobczak, D. J., & Doering, M. (2022). The efficacy and safety of exercise and physical activity on psychosis: A systematic review and meta-analysis. Frontiers in Psychiatry, 13, 807140. doi: 10.3389/fpsyt.2022.807140 36051555 PMC9425642

