# Supplementary material for: Effects of aerobic exercise on hippocampal formation volume in people with schizophrenia – a systematic review and meta-analysis with original data from a randomized-controlled trial
Source: Psychol Med. 2024 Nov 18;54(15):4009–20. doi: 10.1017/S0033291724001867 (PMC11650161; doi:10.1017/S0033291724001867)
Supplement: Roell et al. supplementary material [file S0033291724001867sup001.docx]

**Supplemental Information**

| **Authors** | **N (pre/post)** | | **Attrition** | **Age Mean (SD)** | | **Sex (F/M)** | | **Intervention** | **Control** | **Segmentation** |
| --- | --- | --- | --- | --- | --- | --- | --- | --- | --- | --- |
|  | **Exp** | **Ctrl** | **%** | **Exp** | **Ctrl** | **Exp** | **Ctrl** |  |  |  |
| Pajonk et al.  (2010)  *Germany* | 8/8 | 8/8 | 0/0 | 32.9  (10.6) | 37.4  (8.1) | 0/8 | 0/8 | Cycle ergometry 30 min./3x week/  12 weeks | Table football 30 min./3x week/  12 weeks | Manual (“Analyze” & “SMP99”) |
| Scheewe et al.  (2013)  *Netherlands* | 31/18 | 32/14 | 42/56 | 28.5  (7.3) | 31.1  (8.0) | 4/14 | 2/12 | 1h (40 min. cardio, 20. Min resistance training)/2x week/  6 months | Occupational therapy 1h/2x week/  6 months | Automated (“FIRST” in “FSL”) |
| Lin et al.  (2015)  *Hong Kong* | 46/17 | 46/13 | 63/72 | 24.6  (7.9) | 25.3 (8.1) | 17/0 | 13/0 | Walking, cycling 1h/3x week/  12 weeks | Waitlist | Automated  (“FSL Fast”) |
| Malchow  (2016)  *Germany* | 20/20 | 19/19 | 0/0 | 36.3  (11.7) | 35.3  (14.5) | 6/14 | 6/13 | Cycle ergometry 30 min./3x week/  12 weeks | Table football 30 min./3x week/  12 weeks | Manual  (“Freesurfer”) |
| Khonsari et al.  (2022)  *Iran* | 20/20 | 20/20 | 0/0 | 32.7  (8.6) | 37.2  (7.8) | 8/12 | 11/9 | Cycling, jogging, jumping 30 min./  3x week/8 weeks | Waitlist | NA |
| Roell et al.  (2023)  *Germany* | 44/9 | 47/20 | 79/57 | 34.6  (11.2) | 39.3  (12.1) | 4/5 | 15/5 | Bicycle ergometer 40-50 min./  3x week/6 months | Flexibility, strength training 40-50 min./3x week/6 months | Automated  (“Freesurfer v7.2”) |
| Rosenbaum et al.  (2015)  *Australia* | 5/5 | - | 0/- | 20.2  (4.2) | - | 0/5 | - | Stationary exercise bike 45 min./2x week/12 weeks | - | Automated  (“FIRST” in “FSL”) |
| Dean et al.  (2017)  *USA* | 12/9 | - | 25/- | 19.4  (1.2) | - | 0/9 | - | Bicycle, elliptical machine 30 min./2-3x week/12 weeks | - | Automated  (“Freesurfer v5.3, “FSL”) |
| Woodward et al.  (2018)  *Canada* | 27/17 | - | 37/- | 30.1  (6.9) | - | NA | - | Ergometry, weight training 30 min./3x week/12 weeks | - | Automated  (“Freesurfer v5.3, “FSL”) |

**Table S1.** Detailed patient and study characteristics.

Pre, Baseline; Post, after intervention; Exp, Experimental group; Ctrl, Control group; SD, Standard deviation; F, Female; M, Male; FSL, FMRIN Software Library.

**Risk of bias assessment via RoB 2.0 for between group meta-analysis**

The most recent edition of the Cochrane risk-of-bias tool for randomized trials (RoB 2) was used to assess risk of bias. Scheewe (2012), Lin (2015), and Roell (2023) were deemed to pose high risk of bias, as attrition rates were high (compare D3). Pajonk (2010), Malchow (2016), and Khonsari (2022) posed some concerns, mostly due to failures to thoroughly described randomization processes and adherences to protocols. Overall, it is important to keep in mind that bias is likely to have occurred due high drop-out rates.

**
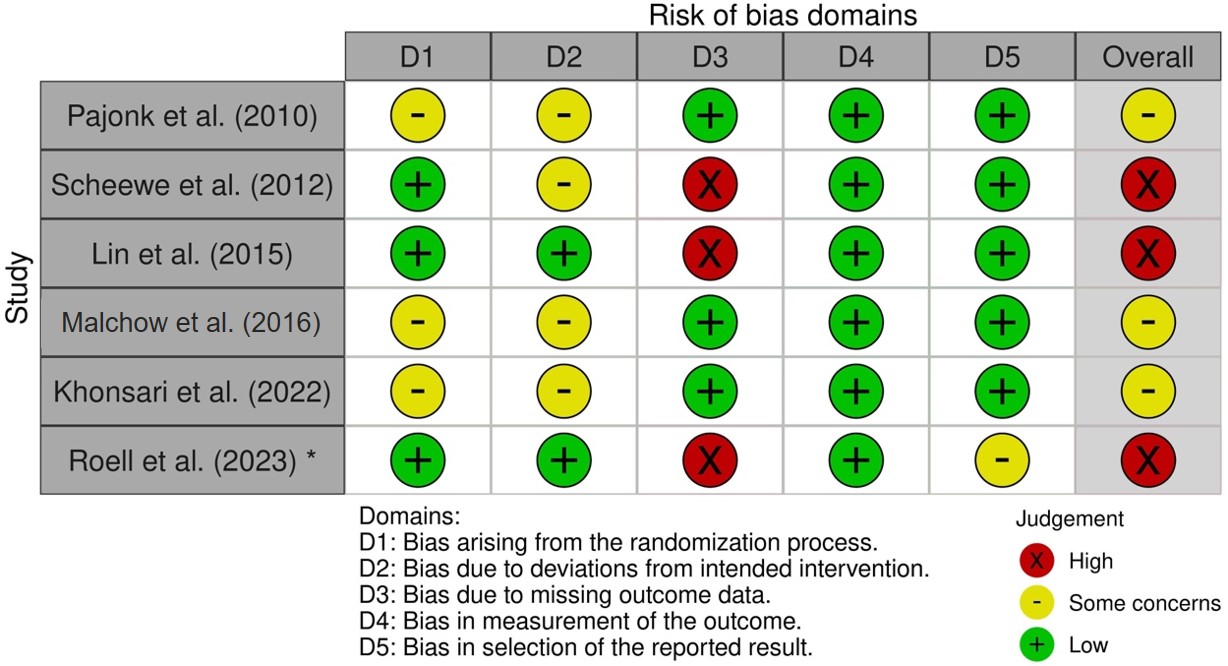
**

**Figure S1.** RoB analysis for meta-analysis between groups. *, ESPRIT study data used in this report

**Risk of bias assessment via RoB 2.0 for within group meta-analysis**

The most recent edition of the Cochrane risk-of-bias tool for randomized trials (RoB 2) was used to assess risk of bias. In addition to previously specified results, Rosenbaum (2015) and Woodward (2018) were also categorized as high risk of bias reports. As others, Woodward (2018) described high attrition rates and Rosenbaum (2015) did not include a control condition. There were some concerns around Dean (2017). Overall, our results should be interpreted with caution, as bias likely occurred in several reports.


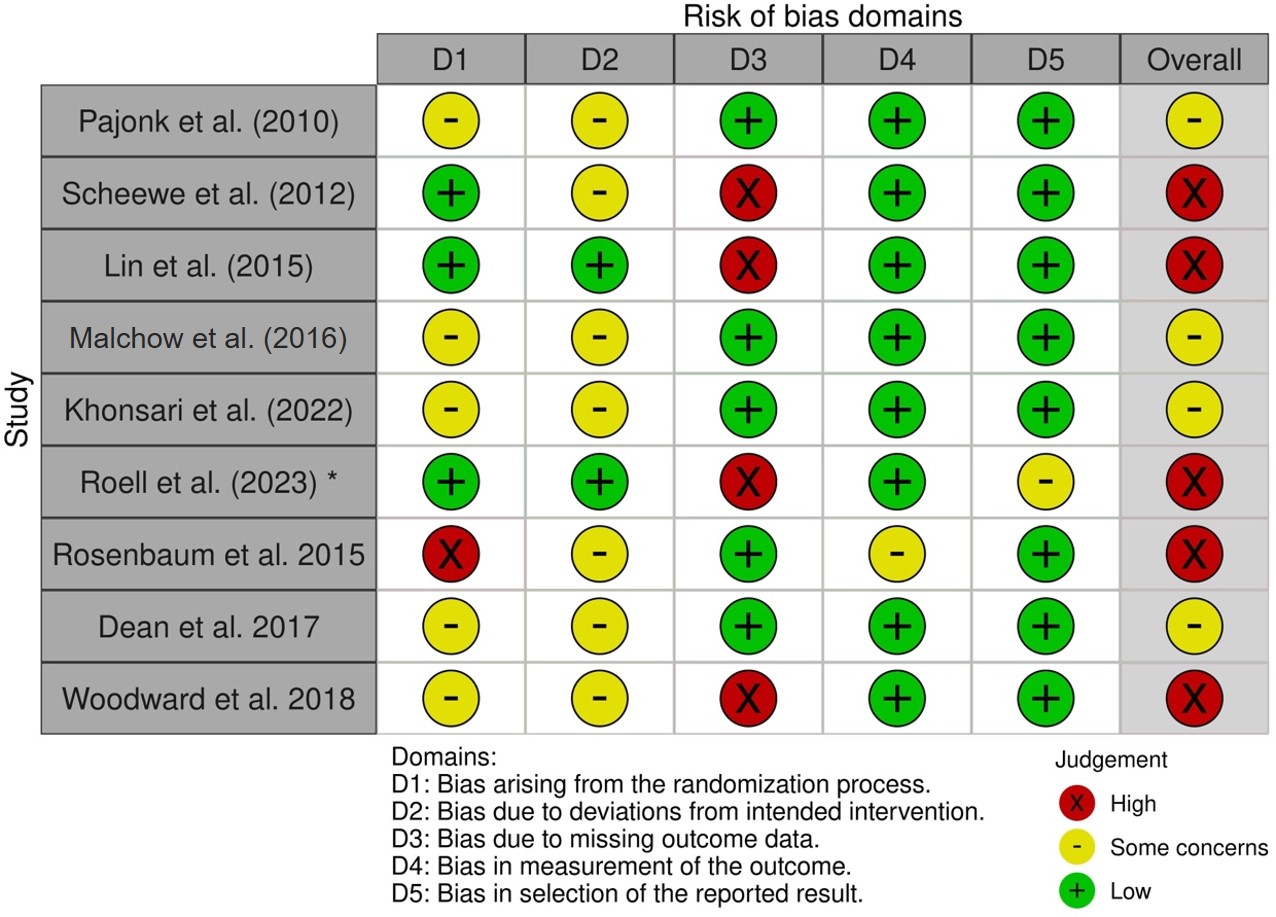


**Figure S2.** RoB analysis for meta-analysis within groups. *, ESPRIT study data used in this report

**Funnel Plot meta-analysis between groups**

**Figure S3.** Funnel plot comparing aerobic exercise and control conditions at post intervention. Circles represent individual studies.

**Funnel Plot within group meta-analysis**

**Figure S4.** Funnel plot comparing pre- and post-intervention scores within the aerobic exercise condition. Circles represent individual studies.

**Distribution of true effects between group meta-analysis**

It is noteworthy to mention that the distribution of true effects is profound, as heterogeneity was substantial in the between groups meta-analysis. Therefore, our conclusions must be carefully interpreted.

**Figure S5**. Distribution of true effects within group meta-analysis.

**Distribution of true effects within group meta-analysis**

Again, the distribution of true effects is profound, as heterogeneity was also deemed to be substantial in the within group meta-analysis. Therefore, our conclusions must be carefully interpreted.

**Figure S6.** Distribution of true effects within group meta-analysis

**Details on the ESPRIT C3 study design, exercise interventions and study sample**

ESPRIT is a study network coordinated from the Central Institute of Mental Health, Mannheim. The present trial is a rater-blind, two-arm, parallel-group, multicenter randomized-controlled clinical trial with an allocation ratio of 1:1 that evaluates the effects of aerobic exercise compared to a flexibility, strengthening and balance training on multiple health outcomes in people with schizophrenia. Involved study sites were the Department of Psychiatry and Psychotherapy of the LMU Hospital in Munich, the Central Institute of Mental Health in Mannheim, as well as the Departments of Psychiatry and Psychotherapy of the University Hospital Charité in Berlin, University Hospital Duesseldorf and University Hospital RWTH in Aachen. The study was conducted in line with the Declaration of Helsinki and was approved by the local ethics committees at each study center.

Participants were enrolled between 22nd July 2016 and 15th December 2021. The intention-to-treat (ITT) sample of the ESPRIT C3 study comprised 180 patients with schizophrenia. Participants were randomly assigned either to the aerobic endurance training (AET) group or to the flexibility, strengthening and balance training (FSBT) group. Randomization was performed by the exercise training staff using a secure web-based randomization system developed by the Institute for Medical Statistics and Computational Biology of the Medical Faculty of the University of Cologne. Permuted blocks of random size stratified by study center were used. The outcome assessors were blinded with regard to the assigned study group.

The subjects in the AET group cycled on a stationary bicycle ergometer at a moderate exercise intensity. A lactate threshold test prior to the intervention allowed to determine the subject-specific wattage to achieve an aerobic metabolic state (approx. 2mmol/L lactate concentration). The training intensity was maintained until the 12th week. After another lactate test, the exercise intensity was adjusted if necessary.

Participants in the FSBT group performed several exercises targeting stretching, mobility, stability, balance, and relaxation, as composed by Liu-Ambrose et al. (Liu-Ambrose et al., 2010). Training intensity was adjusted if the subjects` were able to finish exercises with only low effort. Supervised by a sport scientist, both groups were supposed to exercise up to three times per week between 40 and 50 minutes for six months.

163 patients finished at least one exercise intervention and formed the ITT2 sample. Within the ITT2 sample, 99 subjects gave written informed consent to undergo MRI sessions. MRI data with sufficient quality at baseline and after six months of exercise was available for 29 subjects. Behavioral and neuroimaging data were acquired prior to the onset of the intervention (t0) and six months after the onset of the intervention (t6) by trained study personnel. For further details on the study design and safety, exclusion criteria, ethical approval and informed consent process see Maurus et al. (Maurus et al., 2020).

**MRI data acquisition, processing, and quality control**

MRI data from the study site Munich were acquired in a 3T Siemens Magnetom Skyra MRI scanner. In Mannheim, Berlin, and Aachen, a 3T Siemens Magnetom Tim Trio scanner was used. At each site, a 3D T1-weighted magnetization prepared rapid gradient echo (MP RAGE) with an isotropic spatial was conducted. The sequence parameters are summarized in Table S2.

Quality control of structural images was performed using visual inspection and the automated software MRIQC (Esteban et al., 2017). We rated the image quality manually and computed different sequence-specific quality metrics, such as the signal-to-noise ratio (SNR), contrast-to-noise ratio (CNR), coefficient of joint variation (CJV), entropy focused criterion (EFC), foreground-background energy ratio (FBER), median intensity non-uniformity (INU), Full Width at Half Maximum (FWHM). Images with bad manual quality ratings or with at least one abnormal quality metric were assigned to the categories “exclude” or “need for further inspection”.

Structural images were processed with *recon-all* from FreeSurfer v7.2 and comprised motion correction and averaging (Reuter et al., 2010), removal of non-brain tissue (Ségonne et al., 2004), automated Talairach transformation, segmentation of the subcortical white matter and grey matter volumes (Fischl et al., 2002; Fischl et al., 2004), intensity normalization (Sled et al., 1998), tessellation of the grey matter – white matter boundary, automated topology correction (Fischl et al., 2001; Ségonne et al., 2007) and surface deformation (Dale et al., 1999; Dale et al., 1993; Fischl et al., 2000). Further details on the FreeSurfer pipeline can be found under <http://surfer.nmr.mgh.harvard.edu/>.

The volumes of the HF subfields CA1, CA2/3, CA4, DG and subiculum were computed based on the T1-weighted images using the latest version of the hippocampal module of FreeSurfer v7.2 (Iglesias et al., 2015). The resulting segmentations of the HF were inspected visually to ensure data quality and erroneous cases were excluded. Based on the proportions method (O'Brien et al., 2011), HF volumes were corrected by the intracranial volume across all subjects and sessions.

**Scanning parameters**

**Table S2***.* Scanning parameters.

| **site** | **sequence** | **resolution** | **TR** | **TE** | **TI** | **FA** | **slices** |
| --- | --- | --- | --- | --- | --- | --- | --- |
|  |  |  |  |  |  |  |  |
| Munich | MP-RAGE | 0.8 × 0.8 × 0.8 mm³ | 2060 ms | 2.2 ms | 1040 ms | 12° | 256 |
|  |  |  |  |  |  |  |  |
| Mannheim | MP-RAGE | 1.0 × 1.0 × 1.0 mm³ | 2530 ms | 3.8 ms | 1100 ms | 7° | 176 |
|  |  |  |  |  |  |  |  |
| Berlin | MP-RAGE | 1.0 × 1.0 × 1.0 mm³ | 1570 ms | 2.8 ms | 800 ms | 15° | 176 |

Site, study site; Sequence, type of scanning sequence; FoV, field of view; resolution, voxel size; TR, Time of repetition; TE, echo time; TI, inversion time; FA, flip angle; slices, number of acquired slices; MP-RAGE, T1-weighted magnetization prepared rapid gradient echo; EPI, echo planar imaging.

**Cognitive test batteries**

During the DST-backward the investigator read digit rows of increasing lengths that the subject had to repeat verbally in reverse order. The test was stopped if the participant failed twice in a row at the same level of difficulty. The number of correct trials was counted and z-standardized. The DST-backward targets working memory (DST wm).

Within the VLMT, the investigator read a list of 15 words and the participants had to remember as many words as possible in arbitrary order (VLMT-1^st^). This procedure was repeated five times in a row. After the fifth trial, an interference list of 15 different words was read and the subjects had to name as many words from this new list as possible (VLMT-inter). Thereafter, the subject was asked to remember as many words as possible from the first list (VLMT-6^th^) without repeating it again. After a 20 minutes delay in which other cognitive tests were executed, the participants had to remember as many words as possible from the first list again (VLMT-7^th^). Finally, the investigator reads 50 words including the ones from the first list and interference trial and the subjects had to decide if the corresponding word was part of the first list. The number of correctly remembered or recognized words in each trial was counted and z-standardized. Z-Scores from the first run and the interference trial were averaged to a verbal short-term memory score (VLMT stm), while z-scores from run six and seven were averaged to a verbal long-term memory score (VLMT ltm).

**Test statistics of meta-analysis**

Table S3 summarizes the test statistics of the meta-analysis.

**Table S3.** Test statistics for meta-analyses with primary outcome total hippocampal formation volume

| **Analysis** | **Studies** | **Subjects** | **Meta-analyses** | | | | **Heterogeneity** | | |
| --- | --- | --- | --- | --- | --- | --- | --- | --- | --- |
|  |  |  |  | | | |  | | |
|  | **N** | **n** | **g** | **CI low** | **CI high** | **p** | **Q** | **p** | **I^2^** |
|  |  |  |  |  |  |  |  |  |  |
| Between | 6 | 186 | 0.33 | -0.12 | 0.77 | 0.15 | 11.52 | **0.04*** | 56.58 |
|  |  |  |  |  |  |  |  |  |  |
| Within | 9 | 217 | 0.19 | -0.05 | 0.42 | 0.11 | 23.08 | **0.00**** | 65.43 |

N, number of studies; n, number of subjects; g, effect size Hedges’ g; CI, 95% confidence interval; p, p-value; Q, Cochran’s Q; I^2^, Higgins’ I2; between, meta-analysis of all trials with control group; within, meta-analysis of all clinical trials with and without a control group.

**Clinical relevance of changes in HF subfield volumes**

Table S4 summarizes the test statistics of the multiple linear regressions. Figures S7, S8, and S9 visualize the correlations between subject-specific changes in HF subfield volume and clinical outcomes.

**Table S4***.* Test statistics of the multiple linear regressions.

| **Brain changes**  **(t0 - t6)** | **Clinical changes**  **(t0 - t6)** | **β** | **CI low** | **CI high** | **p_raw_** | **p_fdr_** |
| --- | --- | --- | --- | --- | --- | --- |
| Left CA1 | PANSS positive | 0.02 | -0.93 | 0.98 | 0.959 | 0.987 |
| Right CA1 | PANSS positive | -0.91 | -2.08 | 0.27 | 0.122 | 0.914 |
| Left CA2/3 | PANSS positive | 0.41 | -0.87 | 1.70 | 0.510 | 0.987 |
| Right CA2/3 | PANSS positive | 0.11 | -0.92 | 1.15 | 0.821 | 0.987 |
| Left CA4 | PANSS positive | 0.14 | -0.65 | 0.93 | 0.710 | 0.987 |
| Right CA4 | PANSS positive | -0.07 | -0.81 | 0.67 | 0.848 | 0.987 |
| Left DG | PANSS positive | 0.05 | -0.74 | 0.83 | 0.904 | 0.987 |
| Right DG | PANSS positive | 0.03 | -0.78 | 0.84 | 0.944 | 0.987 |
| Left SUB | PANSS positive | -0.15 | -1.37 | 1.06 | 0.793 | 0.987 |
| Right SUB | PANSS positive | 1.20 | -0.05 | 2.46 | 0.059 | 0.892 |
| Left CA1 | PANSS negative | 0.16 | -0.90 | 1.23 | 0.752 | 0.987 |
| Right CA1 | PANSS negative | 0.14 | -1.25 | 1.53 | 0.838 | 0.987 |
| Left CA2/3 | PANSS negative | -1.39 | -2.67 | -0.10 | **0.036*** | 0.892 |
| Right CA2/3 | PANSS negative | -0.47 | -1.61 | 0.66 | 0.396 | 0.987 |
| Left CA4 | PANSS negative | -0.22 | -1.10 | 0.66 | 0.604 | 0.987 |
| Right CA4 | PANSS negative | -0.06 | -0.88 | 0.76 | 0.880 | 0.987 |
| Left DG | PANSS negative | -0.21 | -1.08 | 0.65 | 0.610 | 0.987 |
| Right DG | PANSS negative | 0.01 | -0.90 | 0.91 | 0.987 | 0.987 |
| Left SUB | PANSS negative | 0.13 | -1.22 | 1.49 | 0.838 | 0.987 |
| Right SUB | PANSS negative | -0.03 | -1.58 | 1.51 | 0.966 | 0.987 |
| Left CA1 | PANSS total | -0.06 | -1.01 | 0.88 | 0.890 | 0.987 |
| Right CA1 | PANSS total | -0.56 | -1.77 | 0.64 | 0.341 | 0.987 |
| Left CA2/3 | PANSS total | -1.01 | -2.20 | 0.19 | 0.093 | 0.914 |
| Right CA2/3 | PANSS total | -0.39 | -1.40 | 0.62 | 0.424 | 0.987 |
| Left CA4 | PANSS total | -0.31 | -1.08 | 0.46 | 0.411 | 0.987 |
| Right CA4 | PANSS total | -0.28 | -1.00 | 0.44 | 0.421 | 0.987 |
| Left DG | PANSS total | -0.36 | -1.11 | 0.39 | 0.330 | 0.987 |
| Right DG | PANSS total | -0.23 | -1.02 | 0.57 | 0.555 | 0.987 |
| Left SUB | PANSS total | -0.27 | -1.47 | 0.92 | 0.636 | 0.987 |
| Right SUB | PANSS total | 0.31 | -1.06 | 1.67 | 0.643 | 0.987 |
| Left CA1 | DST wm | 0.66 | -0.11 | 1.43 | 0.088 | 0.551 |
| Right CA1 | DST wm | 1.05 | 0.12 | 1.99 | **0.029*** | 0.437 |
| Left CA2/3 | DST wm | -0.54 | -2.00 | 0.91 | 0.443 | 0.738 |
| Right CA2/3 | DST wm | -0.22 | -1.95 | 1.51 | 0.791 | 0.841 |
| Left CA4 | DST wm | 0.45 | -0.20 | 1.10 | 0.161 | 0.562 |
| Right CA4 | DST wm | 0.57 | -0.17 | 1.32 | 0.122 | 0.562 |
| Left DG | DST wm | 0.53 | -0.10 | 1.16 | 0.092 | 0.551 |
| Right DG | DST wm | 0.53 | -0.26 | 1.33 | 0.174 | 0.562 |
| Left SUB | DST wm | 0.18 | -1.00 | 1.37 | 0.747 | 0.841 |
| Right SUB | DST wm | -0.26 | -1.44 | 0.92 | 0.646 | 0.839 |
| Left CA1 | VLMT stm | 0.15 | -0.89 | 1.18 | 0.767 | 0.841 |
| Right CA1 | VLMT stm | 0.58 | -0.66 | 1.83 | 0.335 | 0.628 |
| Left CA2/3 | VLMT stm | -1.00 | -2.69 | 0.68 | 0.224 | 0.562 |
| Right CA2/3 | VLMT stm | 0.60 | -1.44 | 2.64 | 0.542 | 0.810 |
| Left CA4 | VLMT stm | -0.13 | -1.01 | 0.76 | 0.767 | 0.841 |
| Right CA4 | VLMT stm | 0.51 | -0.42 | 1.43 | 0.262 | 0.562 |
| Left DG | VLMT stm | -0.10 | -0.96 | 0.77 | 0.813 | 0.841 |
| Right DG | VLMT stm | 0.47 | -0.52 | 1.46 | 0.326 | 0.628 |
| Left SUB | VLMT stm | 0.85 | -0.49 | 2.19 | 0.196 | 0.562 |
| Right SUB | VLMT stm | -0.96 | -2.35 | 0.43 | 0.163 | 0.562 |
| Left CA1 | VLMT ltm | -0.29 | -1.18 | 0.59 | 0.491 | 0.775 |
| Right CA1 | VLMT ltm | 0.05 | -1.06 | 1.16 | 0.925 | 0.925 |
| Left CA2/3 | VLMT ltm | -1.83 | -3.02 | -0.64 | **0.005**** | 0.147 |
| Right CA2/3 | VLMT ltm | -1.60 | -3.18 | -0.01 | **0.049*** | 0.486 |
| Left CA4 | VLMT ltm | -0.41 | -1.15 | 0.33 | 0.253 | 0.562 |
| Right CA4 | VLMT ltm | -0.21 | -1.04 | 0.62 | 0.594 | 0.810 |
| Left DG | VLMT ltm | -0.42 | -1.14 | 0.30 | 0.236 | 0.562 |
| Right DG | VLMT ltm | -0.18 | -1.06 | 0.70 | 0.671 | 0.839 |
| Left SUB | VLMT ltm | -0.31 | -1.53 | 0.90 | 0.591 | 0.810 |
| Right SUB | VLMT ltm | -0.52 | -1.77 | 0.74 | 0.397 | 0.700 |
| Left CA1 | GAF | -0.46 | -1.15 | 0.23 | 0.181 | 0.302 |
| Right CA1 | GAF | -0.73 | -1.64 | 0.18 | 0.109 | 0.271 |
| Left CA2/3 | GAF | -0.65 | -1.60 | 0.31 | 0.174 | 0.302 |
| Right CA2/3 | GAF | -0.74 | -1.45 | -0.03 | **0.041*** | 0.271 |
| Left CA4 | GAF | -0.53 | -1.07 | 0.01 | 0.056 | 0.271 |
| Right CA4 | GAF | -0.43 | -0.95 | 0.09 | 0.097 | 0.271 |
| Left DG | GAF | -0.46 | -1.01 | 0.08 | 0.092 | 0.271 |
| Right DG | GAF | -0.44 | -1.02 | 0.13 | 0.122 | 0.271 |
| Left SUB | GAF | -0.38 | -1.46 | 0.71 | 0.475 | 0.679 |
| Right SUB | GAF | -1.12 | -2.02 | -0.22 | **0.017*** | 0.271 |
| Left CA1 | FROGS | 0.18 | -0.89 | 1.25 | 0.724 | 0.762 |
| Right CA1 | FROGS | -0.28 | -1.70 | 1.13 | 0.675 | 0.762 |
| Left CA2/3 | FROGS | 0.00 | -1.94 | 1.95 | 0.999 | 0.999 |
| Right CA2/3 | FROGS | -1.26 | -3.47 | 0.95 | 0.245 | 0.377 |
| Left CA4 | FROGS | -0.24 | -1.11 | 0.64 | 0.573 | 0.753 |
| Right CA4 | FROGS | -0.79 | -1.72 | 0.15 | 0.093 | 0.271 |
| Left DG | FROGS | -0.22 | -1.09 | 0.65 | 0.602 | 0.753 |
| Right DG | FROGS | -0.73 | -1.73 | 0.28 | 0.146 | 0.292 |
| Left SUB | FROGS | -1.54 | -3.30 | 0.22 | 0.083 | 0.271 |
| Right SUB | FROGS | -0.28 | -1.79 | 1.24 | 0.705 | 0.762 |

CA, cornu ammonis; DG, dentate gyrus; SUB, subiculum; DST, Digit Span Test; VLMT, Verbal Learning and Memory Test; GAF, Global Assessment of Functioning scale; FROGS, Functional Remission of General Schizophrenia scale; PANSS, Positive and Negative Syndrome Scale; β, effect size from multiple linear regression; CI low, lower 95%-confidence interval; CI high, higher 95%-confidence interval; p_fdr_; corrected p-value; p_raw_; uncorrected p-value. Significant results are printed in bold. *, p < 0.05; **, p < 0.01.


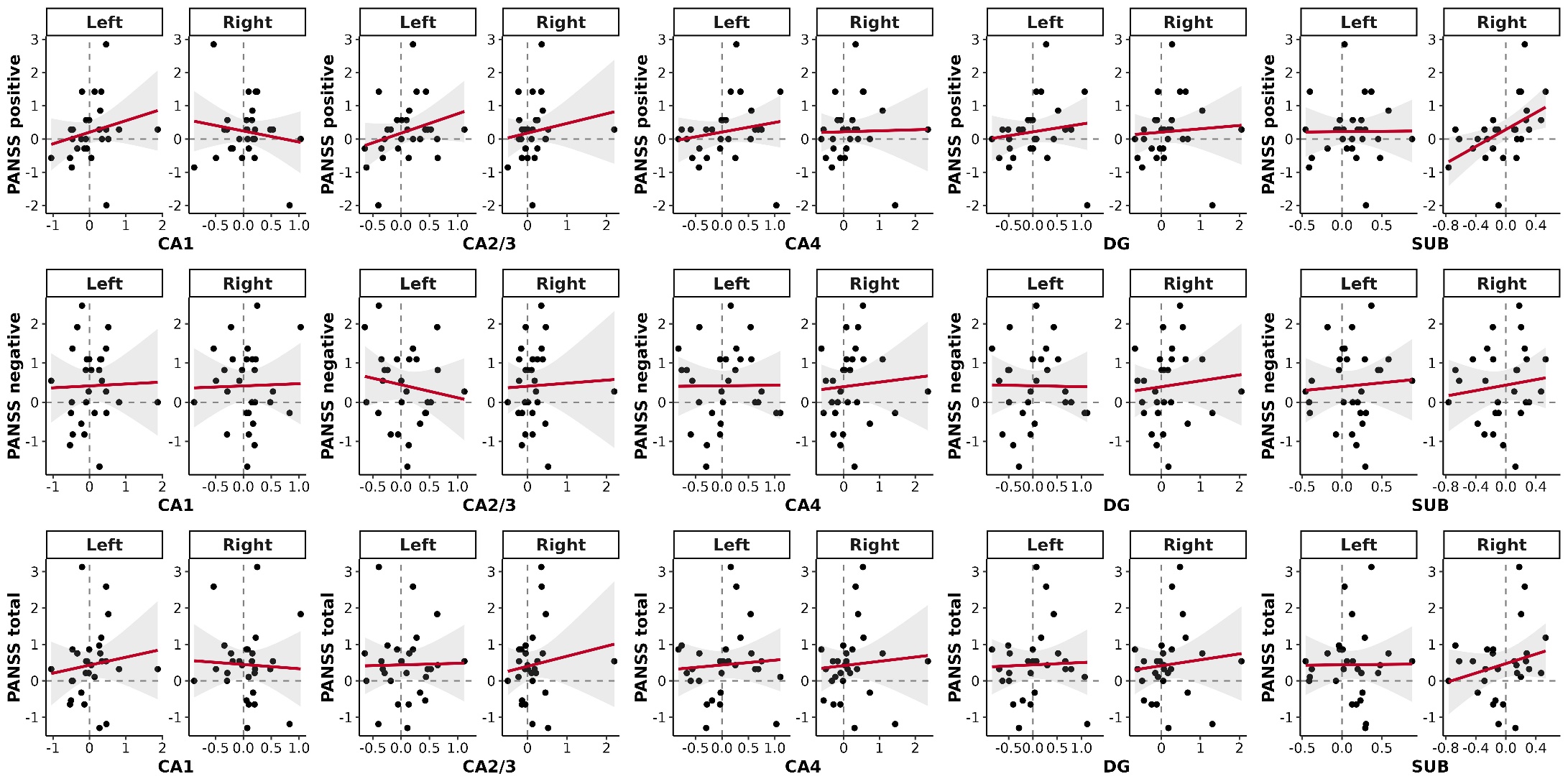


**Figure S7**. Correlations between changes in HF volumes and changes in symptom severity. On the x- and y-axes the corresponding z-standardized difference scores between session t0 and t6 are plotted. A positive link corresponds to an increase in volume and a simultaneous improvement in symptoms. The shadowed area reflects the 95 % confidence interval. CA, cornu ammonis; DG, dentate gyrus; SUB, subiculum; Left, left hemisphere; Right, right hemisphere; PANSS, Positive and Negative Syndrome Scale.


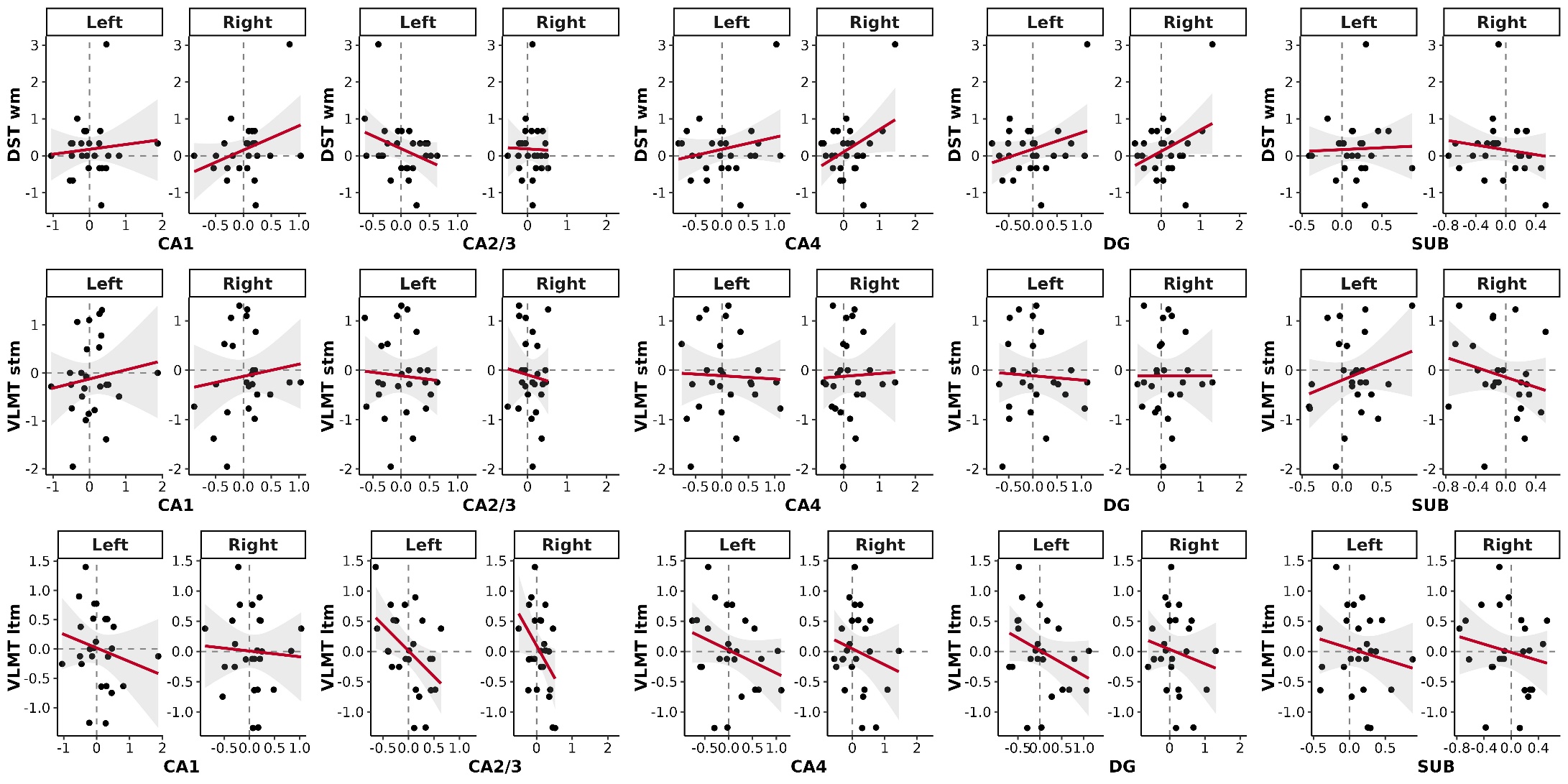


**Figure S8**. Correlations between changes in HF volumes and changes in cognitive functioning. On the x- and y-axes the corresponding z-standardized difference scores between session t0 and t6 are plotted. A positive link corresponds to an increase in volume and a simultaneous improvement in cognition. The shadowed area reflects the 95 % confidence interval. CA, cornu ammonis; DG, dentate gyrus; SUB, subiculum; Left, left hemisphere; Right, right hemisphere; DST wm, Digit Span Test for working memory; VLMT stm, Verbal Learning and Memory Test for short-term memory; VLMT ltm, Verbal Learning and Memory Test for long-term memory.


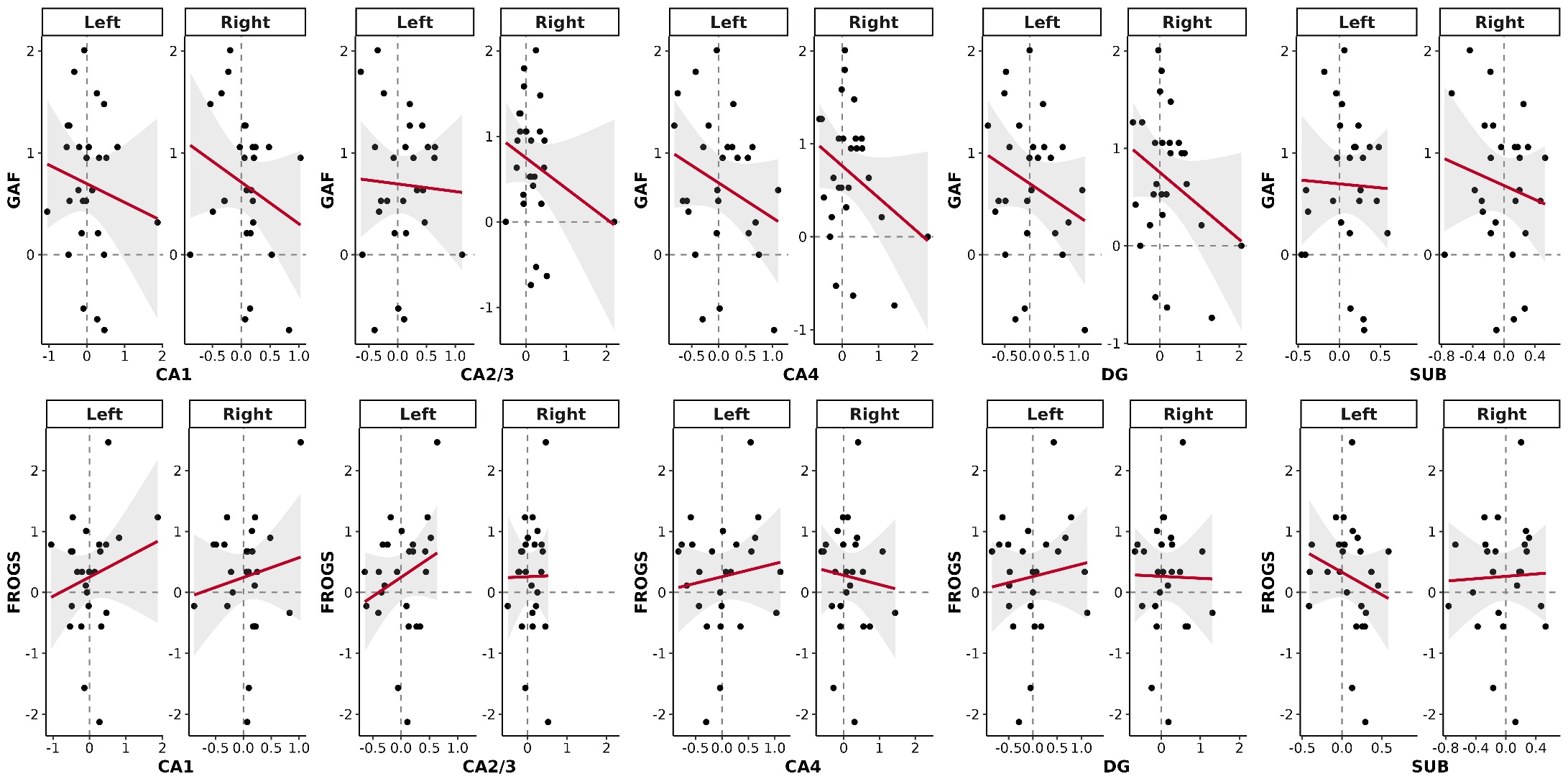


**Figure S9**. Correlations between changes in HF volumes and changes in functioning. On the x- and y-axes the corresponding z-standardized difference scores between session t0 and t6 are plotted. A positive link corresponds to an increase in volume and a simultaneous improvement in functioning. The shadowed area reflects the 95 % confidence interval. CA, cornu ammonis; DG, dentate gyrus; SUB, subiculum; Left, left hemisphere; Right, right hemisphere; GAF, Global Assessment of Functioning scale; FROGS, Functional Remission of General Schizophrenia scale.

**Supplemental References**

Dale, A. M., Fischl, B., & Sereno, M. I. (1999). Cortical surface-based analysis. I. Segmentation and surface reconstruction. *Neuroimage, 9*(2), 179-194. doi:10.1006/nimg.1998.0395

Dale, A. M., & Sereno, M. I. (1993). Improved Localizadon of Cortical Activity by Combining EEG and MEG with MRI Cortical Surface Reconstruction: A Linear Approach. *J Cogn Neurosci, 5*(2), 162-176. doi:10.1162/jocn.1993.5.2.162

Esteban, O., Birman, D., Schaer, M., Koyejo, O. O., Poldrack, R. A., & Gorgolewski, K. J. (2017). MRIQC: Advancing the automatic prediction of image quality in MRI from unseen sites. *PLoS One, 12*(9), e0184661. doi:10.1371/journal.pone.0184661

Fischl, B., & Dale, A. M. (2000). Measuring the thickness of the human cerebral cortex from magnetic resonance images. *PNAS, 97*(20), 11050-11055. doi:10.1073/pnas.200033797

Fischl, B., Liu, A., & Dale, A. M. (2001). Automated manifold surgery: constructing geometrically accurate and topologically correct models of the human cerebral cortex. *IEEE Trans Med Imaging, 20*(1), 70-80. doi:10.1109/42.906426

Fischl, B., Salat, D. H., Busa, E., Albert, M., Dieterich, M., Haselgrove, C., . . . Dale, A. M. (2002). Whole brain segmentation: automated labeling of neuroanatomical structures in the human brain. *Neuron, 33*(3), 341-355. doi:10.1016/s0896-6273(02)00569-x

Fischl, B., Salat, D. H., van der Kouwe, A. J., Makris, N., Ségonne, F., Quinn, B. T., & Dale, A. M. (2004). Sequence-independent segmentation of magnetic resonance images. *Neuroimage, 23*(Suppl 1), S69-S84. doi:10.1016/j.neuroimage.2004.07.016

Iglesias, J. E., Augustinack, J. C., Nguyen, K., Player, C. M., Player, A., Wright, M., . . . Van Leemput, K. (2015). A computational atlas of the hippocampal formation using ex vivo, ultra-high resolution MRI: Application to adaptive segmentation of in vivo MRI. *Neuroimage, 115*, 117-137. doi:10.1016/j.neuroimage.2015.04.042

Liu-Ambrose, T., Nagamatsu, L. S., Graf, P., Beattie, B. L., Ashe, M. C., & Handy, T. C. (2010). Resistance training and executive functions: a 12-month randomized controlled trial. *Arch Intern Med, 170*(2), 170-178. doi:10.1001/archinternmed.2009.494

Maurus, Hasan, A., Schmitt, A., Roeh, A., Keeser, D., Malchow, B., . . . Falkai, P. (2020). Aerobic endurance training to improve cognition and enhance recovery in schizophrenia: design and methodology of a multicenter randomized controlled trial. *Eur Arch Psychiatry Clin Neurosci, 271*, 315-324. doi:10.1007/s00406-020-01175-2

O'Brien, L. M., Ziegler, D. A., Deutsch, C. K., Frazier, J. A., Herbert, M. R., & Locascio, J. J. (2011). Statistical adjustments for brain size in volumetric neuroimaging studies: some practical implications in methods. *Psychiatry Res, 193*(2), 113-122. doi:10.1016/j.pscychresns.2011.01.007

Reuter, M., Rosas, H. D., & Fischl, B. (2010). Highly accurate inverse consistent registration: a robust approach. *Neuroimage, 53*(4), 1181-1196. doi:10.1016/j.neuroimage.2010.07.020

Ségonne, F., Dale, A. M., Busa, E., Glessner, M., Salat, D., Hahn, H. K., & Fischl, B. (2004). A hybrid approach to the skull stripping problem in MRI. *Neuroimage, 22*(3), 1060-1075. doi:10.1016/j.neuroimage.2004.03.032

Ségonne, F., Pacheco, J., & Fischl, B. (2007). Geometrically accurate topology-correction of cortical surfaces using nonseparating loops. *IEEE Trans Med Imaging, 26*(4), 518-529. doi:10.1109/tmi.2006.887364

Sled, J. G., Zijdenbos, A. P., & Evans, A. C. (1998). A nonparametric method for automatic correction of intensity nonuniformity in MRI data. *IEEE Trans Med Imaging, 17*(1), 87-97. doi:10.1109/42.668698
